# Natural approaches for the management of ulcerative colitis: evidence of preclinical and clinical investigations

**DOI:** 10.1007/s13659-024-00463-x

**Published:** 2024-07-30

**Authors:** Rudra Narayan Subudhi, Neelam Poonia, Dilpreet Singh, Vimal Arora

**Affiliations:** Department of Pharmaceutics, University Institute of Pharma Sciences, Chandigarh University, Gharuan, Mohali, Punjab India

**Keywords:** Ulcerative colitis, Pathophysiology, Plant extracts, Essential oils, Nutraceuticals, Phytocompounds

## Abstract

Ulcerative colitis (UC) is a recurring autoimmune disorder characterized by persistent inflammation in the mucosal lining of the lower part of the large intestine. Conventional treatment options such as salicylates, corticosteroids, and immunosuppressants often come with severe side effects, limited bioavailability, and the development of drug resistance, which hampers their therapeutic effectiveness. Therefore, it is imperative to explore natural strategies as safe and alternative treatments for UC. Currently, around 40% of UC patients find relief through natural constituents, which can help reduce toxic side effects and maintain clinical remission. This review aims to provide a summary of both preclinical and clinical evidence supporting the efficacy of various natural substances in the prophylaxis of UC. These natural options include plant extracts, essential oils, nutraceuticals, and phytochemicals. Furthermore, we will delve into the potential mechanisms that underlie the protective and curative actions of these novel herbal agents. In summary, this review will explore the effectiveness of natural remedies for UC, shedding light on their preclinical and clinical findings and the mechanisms behind their therapeutic actions. These alternatives offer hope for improved treatment outcomes and reduced side effects for individuals suffering from this challenging autoimmune condition.

**Graphical Abstract:**

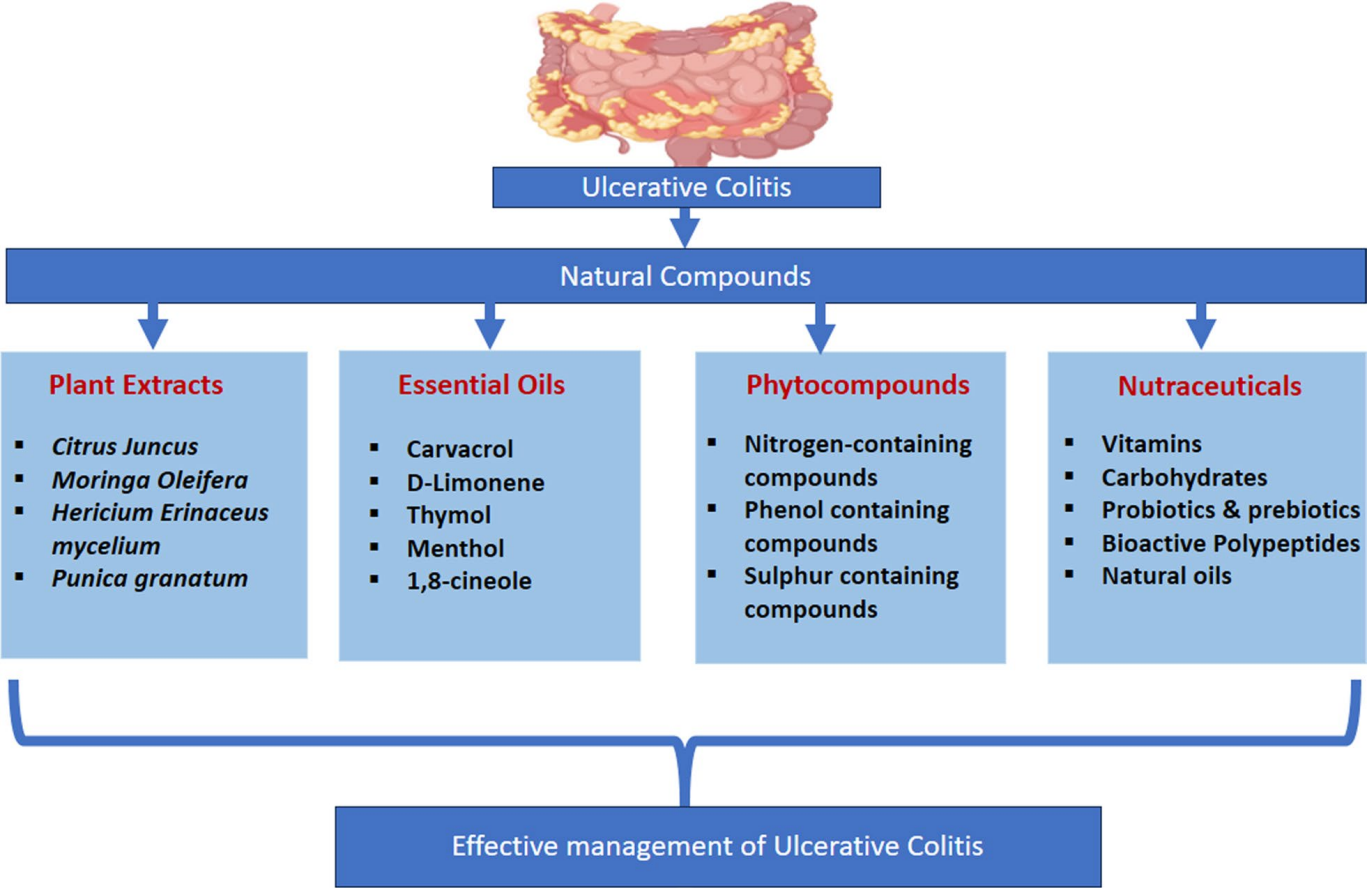

## Introduction

Globally, the prevalence of ulcerative colitis (UC) has seen a significant rise, particularly in Northern Europe and North America, with two distinct age peaks—one between 30 to 40 years and the other occurring in the 5th to 8th decades of life. This condition affects individuals of all genders and its exact cause remains unknown [1, 2]. The increasing incidence of UC is a growing concern in developed countries, such as Europe and the USA, according to epidemiological data [3, 4]. UC, also known as spastic colon, is a persistent auto inflammatory disorder presenting inflammation in the mucosal lining of the large intestine, primarily affecting the distal colon and rectum. It belongs to the group of inflammatory bowel diseases (IBD) and manifests as a chronic inflammatory process in the bowel, marked by periods of clinical relapse and remission [5–7].

The predominant form of IBD is UC, which presents with abdominal pain and rectal bleeding [8]. Severe cases may involve weight loss, tachycardia, fever, blood loss, and bowel enlargement [9]. Various factors, including infectious diseases (such as *C. difficile* and *CMV*), toxic reactions (linked to specific antibiotics and NSAIDs), mesenteric ischemia, or bowel malignancies, can contribute to enteritis and should be ruled out before initiating therapy [10, 11]. Patients taking immunosuppressants or steroids should also be screened for opportunistic UC include hematochezia (bloody stools), fecal incontinence, rectal tenesmus (the urge to defecate), and varying degrees of abdominal cramps and pain that are relieved after defecation [12, 13]. While the exact cause of UC remains elusive, recent research has pointed to factors such as abnormal immune responses, intestinal dysbiosis, environmental influences, and genetic susceptibility as potential contributors [14].

In recent decades, numerous investigations demonstrated the protective, medicinal, preventive, and mitigating effects of natural compounds on colonic inflammation [15, 16]. Currently, 40% of UC patients have found relief through the use of natural compounds, which not only reduce toxic side effects but also help maintain clinical remission [17]. These natural compounds often possess oxido-inflammatory and immunomodulatory activities, rendering them potential candidates for the treatment of UC [18, 19]. Therefore, in this article, we have provided an insight into the importance of natural or herbal substances; including plant extracts essential oils, nutraceuticals, and phytochemicals, along with the underlying mechanisms that contribute to their efficacy in preventing or treating UC. Various clinical trials using natural compounds in management of UC have been summarized in Table 1.

**Table 1:** Clinical trials related to herbal drugs for the prophylaxis of Ulcerative colitis

S. No	Herbs Used	Type of study	Design	Trial No	Patients	Phase	Date
1	Pomegranate Juice Ellagitannins	Interventional	Quadruple Randomized	NCT03000101	18	NA	2023-08-30
2	Red Marine Algae	Interventional	Triple Randomized	NCT03869905	40	2	2023-04-04
3	Probiotic	Interventional	Single-site randomized double-blind placebo-controlled trial	NCT03266484	100	NA	2023-03-14
4	Lactobacillus Rhamnosus GG	Interventional	Triple Randomized	NCT04102852	76	1, 2	2022-10-26
5	β-fructans	Interventional	Double Randomized	NCT02865707	89	NA	2022-04-14
6	Probiotic	interventional	Double-blind randomized clinical trial	NCT04223479	30	NA	2022-03-01
7	Saffron	Interventional	Double Randomized	NCT04749576	100	NA	2021-02-11
8	Agaricus Blazei Murill	Interventional	Single Randomized	NCT01496053	100	2, 3	2020-10-08
9	Probiotics VSL#3®	Interventional	Randomized, double-blind, placebo-controlled	NCT03415711	14	NA	2020-01-18
10	Mango Polyphenolics	Interventional	Single Group Assignment	NCT02227602	20	NA	2019-12-04
11	Gegen Qinlian Decoction	Interventional	Randomized	NCT04057547	60	1	2019-10-15
12	Multistrain Probiotics	Interventional	Double Randomized	NCT04006977	60	NA	2019-09-12
13	Lactobacillus Reuteri	Interventional	Double-blind randomized clinical trial	NCT03798210	40	2	2019-01-14
14	Cannabinoid Receptor	Observational		NCT02735941	90	NA	2018-07-30
15	Berberine	Interventional	Randomized, open-label, Phase IV Clinical Trial	NCT02962245	238	4	2016-11-11
16	Prebiotics	Interventional	Double blind randomized placebo-controlled trial	NCT02865707	89	NA	2016-08-12
17	Curcumin	Interventional	Randomized, Double-blind	NCT01320436	50	NA	2016-02-12
18	Dietary Oats	Interventional	Controlled Study, Triple randomized	NCT00751933	36	2	2015-04-10
19	Aloe Barbadensis Miller	Interventional	Double Randomized	NCT01783119	60	0	2013-02-04
20	Coltect (curcumin, green tea and selenomethionine)	Interventional	Randomized	NCT00793130	30	NA	2008-11-19

## Materials and methods

This study was performed by reviewing extensive details from preclinical and clinical investigations on Natural Compounds in managing Ulcerative Colitis. A comprehensive approach was adopted, utilizing international scientific databases like PubMed, Scopus, and specialized resources such as ClinicalTrials.gov to assimilate the latest research findings and clinical trial data.

## Pathology of UC

UC progression is attributed to the erosion of mucosal barriers integrity, influenced by environmental and genetic factors, as well as abnormal immune responses that modify complex proteins within tight junctions, leading to increased apoptosis [20, 21]. Human epithelial homeostasis relies on a symbiotic flora, and any disruption in the composition of the intestinal microecosystem can trigger inflammatory responses. In this context, the delicate balance of the gut microbiota plays a crucial role in maintaining mucosal integrity. In UC patients, there is a decline in beneficial bacteria that aid in intestinal mucosa healing, coupled with an increase in the permeability of their intestinal epithelial barriers [22, 23].

**Fig. 1:**
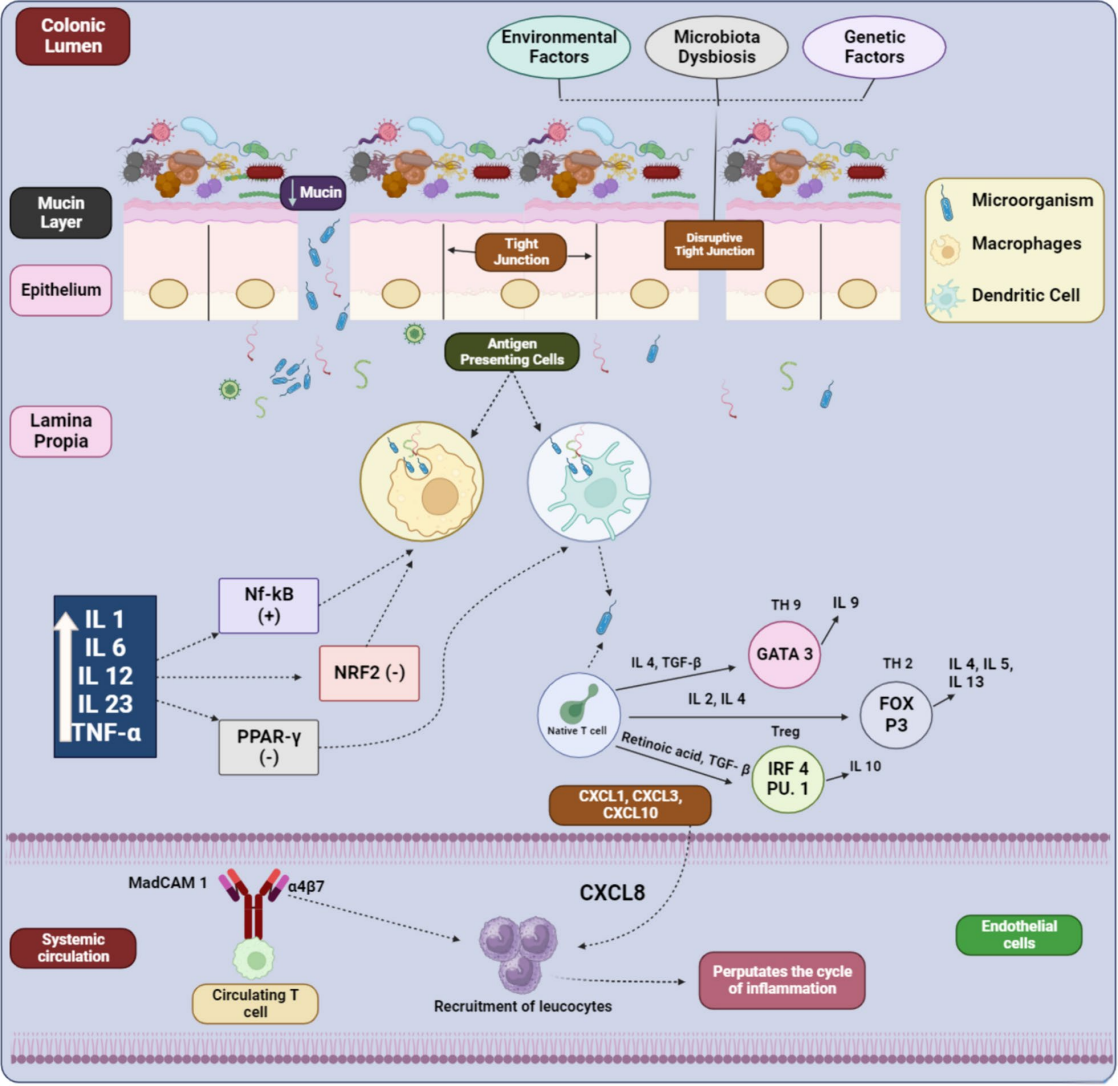
Schematic illustration of pathogenic mechanisms of UC (Created by BioRender)

Local and systemic cytokines viz*. *TNF, IFN, and IL-13, produced by NK-cells and helper T-cells, compromise the integrity of tight junctions, resulting in the destruction of this protein complex (Fig. 1). Consequently, an increase in intestinal permeability occurs, allowing more intraluminal antigens to enter and induce inflammatory responses [24, 25].

Antigen-presenting cells (macrophages and dendritic cells), are activated by symbiotic flora through toll-like receptors [26, 27]. The activation of dendritic cells triggers the differentiation of CD4+ T cells into various effector T cell subsets, viz. Th2, Th9, and regulatory T cells (Treg), influenced by IL-2, IL-4, TGF-beta, and retinoic acid [28, 29]. This immune activation leads to a persistent inflammatory state within the colonic mucosa, contributing to the chronic nature of UC. Moreover, the dominance of FOX P3, GATA binding protein 3 (GATA3), and IRF4 PU.1 in the lamina propia of UC patients promotes inflammation. Consequently, CD4+ T cells in the colonic mucosa are considered a key element in the etiology of UC [30, 31].

The continuous recruitment and activation of immune cells result in the release of various pro-inflammatory mediators, perpetuating the cycle of inflammation and tissue damage. In the luminal epithelium, macrophages play a crucial role as principal effector cells, which is vital for maintaining immune system and intestinal mucosal homeostasis [32, 33]. Activation of macrophages, in turn dysregulates the immune-inflammatory pathways viz*.* nuclear factor transcription and inflammasome pathways, which induces oxidative stress and inflammation [34, 35]. This sustained inflammatory response leads to the formation of ulcers and erosion of the colonic mucosa, characteristic of UC pathology.

## Diagnosis of UC

The diagnosis of UC involves a multifaceted approach, wherein clinical evaluation, a comprehensive medical history assessment, and diagnostic tests are done [36]. It typically begins with a detailed discussion of the medical history, during which the healthcare provider will inquire about the symptoms, their duration, and any relevant family history of inflammatory bowel diseases. Following this, a comprehensive physical examination is conducted, aimed at identifying signs of inflammation, abdominal tenderness, and any other physical findings that may suggest UC [37, 38]. Laboratory tests play a significant role in the diagnostic process. Blood tests, such as CBC, ESR, and CRPare often carried out to evaluate indicators of inflammation [39]. Stool samples are analyzed to rule out infections or other gastrointestinal conditions that can present with symptoms similar to UC [40]. These tests help in differentiating UC from other potential causes of similar clinical presentations.

Imaging studies can also be helpful in diagnosis [41]. A key procedure is a colonoscopy, where endoscope (flexible tube with a camera) is introduced through the rectum and advanced via colon. This enables direct visualization of the colon and rectum's lining, allowing the physician to identify signs of inflammation and ulcerations, as well as to take biopsies for further examination [42, 43]. This visual assessment is crucial for confirming UC and assessing its severity. Biopsies are crucial for confirming the diagnosis of UC and distinguishing it from other gastrointestinal conditions. During endoscopy, small tissue samples are taken from inflamed areas of the colon and rectum. These biopsies are then sent to a pathology laboratory for microscopic evaluation [44, 45]. A flexible sigmoidoscopy is a similar procedure, but it focuses on the lower part of the colon (sigmoid colon) and the rectum. In some instances, an abdominal X-ray or a CT scan may be used to assess the extent and severity of inflammation [46].

Differential diagnosis is a critical step as UC shares symptoms with other conditions like infectious colitis, Crohn’s disease or IBS (irritable bowel syndrome). Serological markers and fecal calprotectin levels can further assist in distinguishing UC from these other conditions. Careful evaluation, along with a combination of clinical assessment and diagnostic tests, helps differentiate UC from these conditions. Following a confirmed diagnosis, healthcare providers assess the extent and severity of UC, which guides treatment decisions. This assessment may involve using established scoring systems such as the Mayo Score or UCDAI (Ulcerative Colitis Disease Activity Index). Regular follow-up appointments and monitoring are crucial for evaluating disease progression, response to treatment, and the potential development of complications [47, 48].

In conclusion, diagnosing UC is a complex process that requires collaboration among various medical professionals, including gastroenterologists, radiologists, and pathologists. An early and precise diagnosis is paramount for effective management and improving the patient's lifestyle. Multidisciplinary care teams play a vital role in ensuring comprehensive evaluation and ongoing management of UC. Treatment strategies may differ based on disease extent and severity, depicting the importance of an accurate diagnosis for tailoring an appropriate management plan [49, 50].

## Present clinical regimens of UC

**Fig. 2:**
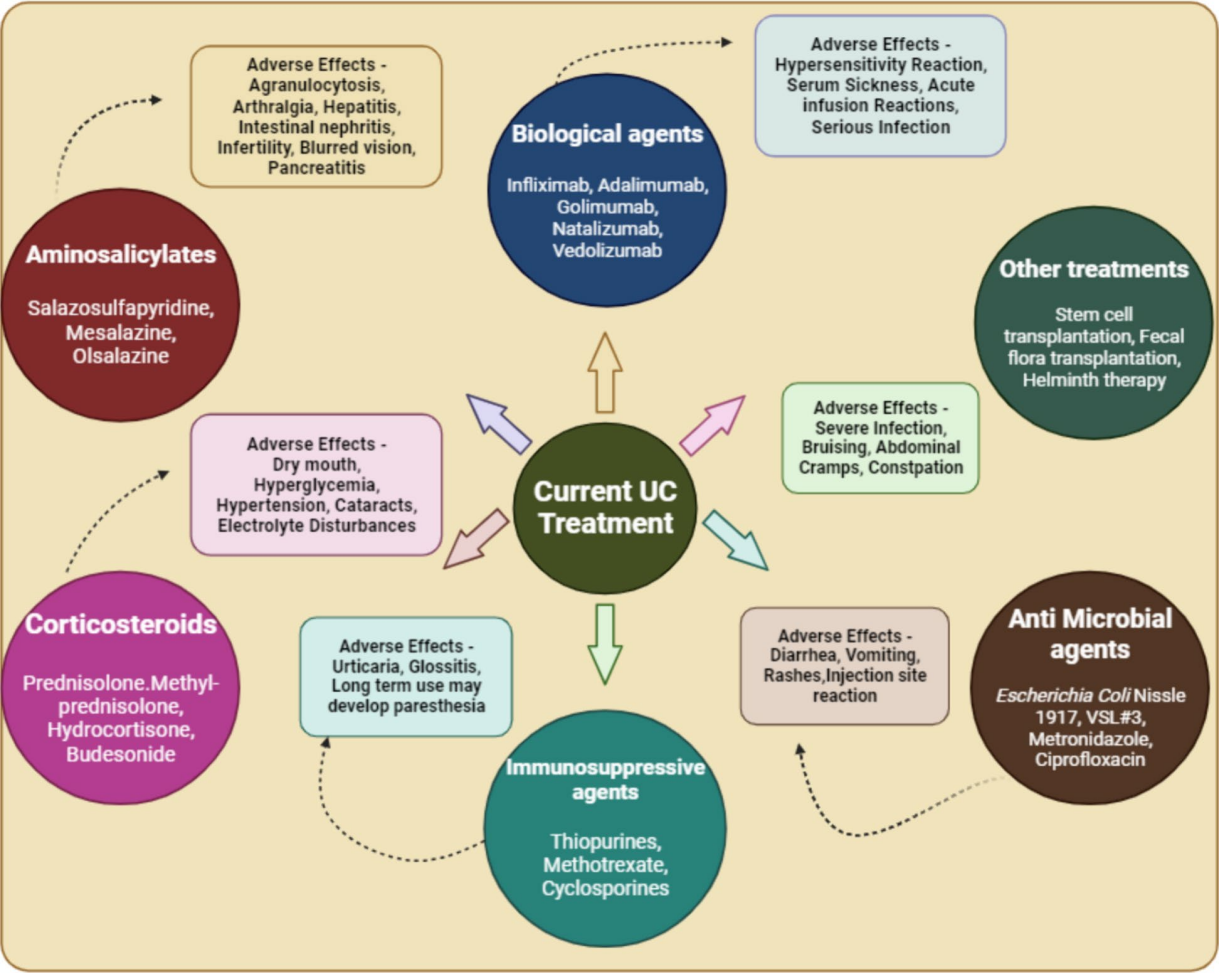
Current recommended treatments for UC along with their drawbacks (Created by BioRender)

Treatment regimens for UC aim for inducing and maintaining clinical remission as well as to prevent long term complications such as colorectal cancer, disability and colectomy. Main objective of remission is to resolve the clinical symptoms which include amelioration of bowel movements, stoppage of rectal bleeding and mucosal healing [51]. Present clinical regimens for UC typically involve a combination of medication, lifestyle modifications, and sometimes surgery. The chief treatment options for UC are aminosalicylates, calcineurin inhibitors, immunomodulators, Janus kinase (JAK) inhibitors and corticosteroids (Fig. 2) [52, 53]. However, these regimens have shortcomings like medication associated issues (susceptibility to infections, liver problems, bone density loss, and allergic reactions), limited efficacy (frequent flare-ups), biologic therapy risks (infusion reactions, may reduce the effectiveness of antibodies), surgery considerations (may cause potential complications), cost and accessibility (biologics and JAK inhibitors), psychological impact etc. [54–56].

## Novel herbal approaches for UC

Novel herbal approaches for UC are gaining huge attention as complementary or alternative therapies to conventional medications [57]. Numerous herbs and natural compounds have demonstrated promising ‘antioxidant, anti-inflammatory and immunomodulatory’ actions both in preclinical and clinical studies [58]. These days’ researchers are exploring bioactive compounds from botanical sources, such as dietary agents, plant extracts, essential oils, nutraceuticals and isolated phytochemicals, which possess additional immune modulating properties for the management of UC. While further research is necessary to establish their safety and effectiveness, these herbal approaches offer a promising avenue for individuals seeking alternative or adjunctive treatments for UC [59, 60].

### Mechanisms underlying treatment of UC

The treatment of UC involves the modulation of various immunoinflammatory pathways, including the activation and suppression of key mechanisms such as PPARγ, Nrf2, NF-kB, and macrophages, along with the regulation of the Th17/Treg balance [61]. These multifaceted processes play pivotal roles in ameliorating the pathogenesis of UC.

Targeting on the regulation of Th17/Treg cells to precise their imbalances is an effective strategy to prevent the occurrence of UC. Many researchers have reported that monoclonal antibodies blocks or inhibits the Th17 cell differentiation effectively reducing the colonic inflammation by targeting various receptors or pathways. Considering the high cost of antibodies, present research has been focused on natural constituents with pharmacological activities that can also modulate Th17/Treg imbalances by regulating “transcription factors (RORγt, STAT3, Foxp3 and STAT5); the signal molecules (mTOR, PI3K/Akt, AMPK, Nrf-2/HO-1, HIF-1α, Notch, RhoA and GSK-3β); and the receptors (AhR, ERβ, PPARγ and TNFR2)” [62–64]. These natural constituents offer a cost-effective and accessible means of targeting the immune system to alleviate UC symptoms. Studies have shown plant products activates PPARγ factors by inhibiting activation of MAPK, adhesion marker-1 and E-selectin expressions, thereby reducing colonic inflammation [65–67]. Herbal products can be used as therapeutic adjuvants in UC and colorectal cancer associated with UC as they can regulate Nrf2 and, thereby reduces colonic inflammation via regulating various genes involved in cellular redox, protein degradation, DNA repair, xenobiotic metabolism, and apoptosis. They act by inhibiting the NF-kB activation pathways [68, 69]. As NF-kB activation plays a major role in the occurrence and development of UC by activating the release of proinflammatory cytokines. The modulatory mechanisms involved in NF-kB regulation by phytochemicals result in reduction of TNF-α, IL-1β, IL-6, IFN-γ, and COX-2 levels and augmented occludin, claudin-1, zonula occludens-1, and IL-10 expression. Thus, herbals by inhibiting of NF-kB activation pathway at the first stage, positively affects the progression of disease [70, 71]. Since, the activation of macrophages by extracellular pathogens leads to the activation of NF-KB and PPAR gamma, resulting in the release of inflammatory mediators inside the mucosa, leading to the development of the disease. Therefore, the main target to inhibit the progression of UC is to transfer the polarization of macrophage in to M2 phenotype. The related modulatory mechanisms namely regulating Hadhb-mediated FAO, the Sirt1/NF-kB signaling pathway, and mTORC2/PPAR-g signaling and other pathways are involved [72, 73]. This comprehensive understanding of the underlying mechanisms allows for the development of targeted herbal therapies that can more effectively manage UC.

In summary, the integration of novel herbal approaches into the treatment plan for UC offers a multifaceted strategy that addresses the complex immune-inflammatory pathways involved in the disease. These approaches provide a promising avenue for enhancing the efficacy of existing treatments while minimizing side effects and improving patient outcomes.

### Plants

Natural products such as plant extracts have shown therapeutic, preventive as well as protective effect against UC in various chemically induced animal models (Table 2). Plant extracts act by multiple defense mechanisms via suppression of inflammatory biomarkers, elevation of anti-oxidant enzyme expressions in the colon tissue or by maintaining the homeostasis of epithelium [74].

**Table 2:** Various extracts and their potential benefits for the management of Ulcerative colitis

Sl No	Plant name	Parts of plant used	Active compounds	Mechanism of ulcer healing	Dose	In vitro/In vivo models used	References
1	*Zanthoxylum alatum*	Seed	Umbelliferone, Quercetin, Resveratol-3-O-glucoside, Benzoic acid	Inhibited the NF-κB translocation Regulated the release of free cortisol Reduced protein expression Increased the antioxidant markers	200 mg/kg	HEK293 cells and DSS-treatedUC model of mice	[89]
2	*Citrus junos*	Seed	Narirutin, Hesperetin7-(2, 6-dirhamnosylglucoside, Naringin, Hesperidin, Neohesperidin	Suppressed TNF-α Inhibited NF-κB pathway activation	50 g/kg	LPS stimulated mice and DSS treated UC model of mouse	[75]
		Seed	Methyl sudachinoid A, Ichangensin-type Limonoids	Down regulated proinflammatory cytokines Supressed NF-κB pathway	NA	LPS-induced RAW 264.7 macrophages of mouse and HT-29 human cells of colon epithelium	[76]
		Peel	Flavanoids	Reduced expression of COX-2 and p38 phosphorylation Inhibited tumour growth in tumor Xenograft model	100 mg/(kg day)	LPS induced RAW264.7 macrophage and DSS treated UC model of mice	[77]
3	*Mimosa pudica*	Leaf	Flavanoids and phenolics	Inhibited oxidative and inflammatory markers	400 mg/kg	A.A treated UC model of rat	[90]
4	*Moringa oleifera*	Seed	Moringa isothiocyanate-1	Upregulated Nrf2-mediated enzymes Decreased proinflammatory biomarkers	150 mg/kg	DSS treated acute and chronic model	[78]
		Leaf	Moringa oleifera leaf polysaccharides	Down-regulated the activated pathways viz*.* MyD88, TLR4, NF-κB etc Up-regulated the PPAR-γ and mRNA expression of IL-10	25, 50, and 100 mg/kg/day	DSS treated UC model of mice	[79]
		Seed	Moringa oleifera pepetides	Inhibited the JAK-STAT pathway activation Modulated gut microbiota dysbiosis	200, 400, 800 mg/kg	DSS induced colitis model of mice	[80]
		Leaf	Phenolics	Down-regulated protein expression of NF-κB p65 &p-IκBα Attenuated cell infiltrations Inhibited inflammatory markers in the colon tissue	200 mg/kg	DSS treated UC model of mice	[81]
5	*Allium sativum*	Clove	Flavanoids, phenolics and organosulfur compounds	Suppressed NF-kB activation Inhibited COX-2 and gene expressions in mouse colon	1–100 µg/mL	LPS-induced mouse colon specimen (Ex vivo)	[91]
6	*Cordia vignei*	Leaf	Flavanoids, alkaloids, saponins	Decreased pro- inflammatory markers Increased pro-oxidant markers	30–300 mg/kg	A.A treated UC model of rat	[92]
7	*Alstonia boonei De Wild*	Stem bark	Tannins, saponins, alkaloids, steroids, flavonoids and phenols	Inhibited TNF-α, PGE2, IL-6& IL-1β production in epithelial colonic cells Reduced malondialdehyde and NO levels	125, 250 mg/kg	DSS treated UC model of rat	[93]
8	*Hericium erinaceus mycelium*	Mycelium	EP-1 polysaccharide	Suppressed expression of GPR41 and GPR43 in colon tissues Increased short chain fatty acids	0.6 and 1.2 g/kg	A.A treated UC model of rat	[82]
		Mycelium	EP-1 polysaccharide	Reduced apoptosis Ameliorated mitochondrial function	1.2 and 2.5 g/kg	Caco-2 cells &A.A treated UC model of rat	[83]
		Mycelium	Low weight polysaccharide (HEP10)	Inhibited immunoinflammatory pathways viz*.*NLRP3, MAPK, and NF-κB etc Suppressed the secretion of inflammatory markers viz*.*inducible iNOS, and COX-2 etc	50, 100, 200 mg/kg	LPS induced murine macrophage RAW264.7 cells and DSS treated UC model of mice	[84]
		Mycelium	Polysaccharides	Inactivated NF-κB pathway Declined the levels of NO, malondialdehyde, myeloperoxidase and other inflammatory markers	250 and 500 mg/kg/day	TNBS treated UC model of rat	[85]
	*Hericium erinaceus*, *berberine*, *and quercetin*, *biotin*, *and niacin*	Mycelium	Polysaccharides, flavanoids, alkaloids, phenolics	Increased expressions viz mRNA and proteins etc Decreased the proinflammatory cytokines Suppressed the expression of-TNF-alpha and COX-2	NA	Tissue specimens from inflammed mucosa (Ex vivo)	[86]
9	*Punica granatum*	Fruit	Punicalagin (PW), *P. granutum* juice (PJ)	Suppressed NF-κβ pathway activation Reduced pro inflammatory cytokine levels	P.granutum juice (400 mg/kg), Punicalagin (4 mg/kg)	2, 4-dinitrobenzene sulfonic acid treated UC model of rats	[87]
		Peel	Ellagic acid	Reduced Lichtiger Colitis Activity Index score Inactivated NF-κβ signaling pathway Decreased inflammatory biomarkers in the colon	6 g dry peel/day	Randomized, placebo controlled clinical trial	[88]
10	*Rubia cordifolia*	Aerial part	Flavanoids and steroids,	Inhibited formation of NLRP3 inflammasome & signal transducer pathways Suppressed release of inflammatory cytokines Reduced macrophage cell infiltration	250 and 500 mg/kg	DSS treated UC model of mice	[94]
11	*Sesbania grandiflora*	Leaf	Polyphenol, flavonoid and flavanone	Inhibited TLR4 receptor mediated inflammation Inhibited free fatty acid levels Restored the levels of anti-oxidant biomarkers	200 mg/kg	A.A treated UC model of mice	[95]
12	*Tagetes erecta L*	Flower	Lutein	Reduced inflammatory cytokines secretion	30–300 mg/kg	DSS treated UC model	[96]
13	*Ziziphus spina-christi*	Fruit	Polyphenolics and flavanoids	Exhibited anti-oxidant and free radial scavenging activity Possessed strong anti-inflammatory activity decreasing pro-inflammatory cytokines Inhibited apoptosis	100, 200, and 400 mg/kg	A.A treated UC model of rat	[97]
14	*Banxia Xiexin*	NA	Quercetin, baicalein, wogonin, and baicalin	Suppressed the expression of COX-2, pro inflammatory cytokines Inhibited the activation of NF-κBp65 Nrf2 expression & SOD activity were elevated	8.7 g/kg	DSS treated UC model of mice	[99]
15	*Averrhoa bilimbi L*	Fruit	Oxalic acid and Vitamin C	Decreased COX-2 expression Regulated the pathological markers in the colon tissue Enhanced the anti-oxidant markers in colon	50 mg/kg/bwt and 100 mg/kg/bwt	A.A treated UC model of rats	[98]

#### *Citrus* junos

*Citrus junos*, commonly known as Yuzu and part of the Rutaceae family, is thought to possess anti-colitic properties. Numerous studies have indicated its potential advantages in mitigating intestinal inflammation. For instance, Abe et al*.*, 2018 investigated the efficacy of Yuzu (*Citrus junos*) extract in a murine model induced with DSS (dextran sulphate sodium). They assessed the oxido-inflammatory balance in mice stimulated with lipids in vivo. The findings revealed a significant suppression of the macrophage cell line and proinflammatory cytokines, via inhibition of the NK pathway suggesting that the peel extract holds promise for mitigating the symptoms associated with UC [75]. In 2020, Mina Lee and colleagues isolated 13 limonoids from Citrus junos seed extract, among which sudachinoid A, a novel compound, and Ichangensin showed promise in treating UC. Researchers observed the anti-inflammatory efficacy of these phytocompounds, employing ELISA and Western blot analysis on mouse macrophages via downregulation of pro-inflammatory cytokines [76]. Another study demonstrated beneficial effects of Yuzu extract via inhibition of cox-2 and suppression of monocyte chemotactic protein, TNF, interleukins, p38 phosphorylation and nitric oxide synthase in RAW264.7 cells. Additionally, the positive outcomes were consistent in a murine colitis model, where histological examinations revealed a notable inhibition of disease activity index (DAI) in the treatment groups that received Yuzu peels extract. These promising results suggest the potential therapeutic efficacy of Yuzu extract in mitigating inflammation and supporting the management of colitis [77].

#### *Moringa oleifera*

*Moringa oleifera*, a member of the Moringaceae family, is renowned for its diverse medicinal properties effective against numerous ailments. Its anti-inflammatory properties have shown to mitigate inflammation and modulate gut microbiota homeostasis. For example, an investigation performed by Youjin et al. investigated the oxidoinflammatory mechanisms of isothiocyanate isolated from *M. oleifera* seed extract both in acute and chronic ulcer models. Utilizing DSS aggravated UC models, the research outcomes revealed a significant upregulation of antioxidant markers and epithelial barrier proteins, along with the inhibition of inflammatory keratinocyte-derived cytokines and nitric oxide levels. Importantly, the results demonstrated that this extract exerts its therapeutic effects by activating Nrf2 signaling pathways. This activation subsequently led to the suppression of interleukins, inducible nitric oxide synthase (NO), fecal lipocalin-2, and the upregulation of GSTP-1, claudin-1, and ZO-1 in the intestine. These results highlight valuable insights into the complex molecular mechanisms through which this extract mitigates inflammation and oxidative stress in ulcerative conditions [78]. Husien et al., conducted a study to investigate the underlying mechanisms of polysaccharides from *M. oleifera* by assessing inflammatory scores, DAI, tight junction proteins, interleukins, and mRNA expressions in the colonic mucosa in a mouse model induced with DSS. Results provided evidence for the preventive actions of this extract by activation of PPAR-γ and downregulation of TLR-4 pathway activation [79]. Hong et al. demonstrated the anticolitic activity of an isolated peptide from *M. oleifera* in a DSS-aggravated colitis model. Through a comprehensive assessment using RNA sequencing, the study revealed that peptide treatment exhibited the ability to inhibit microbial dysbiosis and the JAK-STAT activation pathway. This inhibition, in turn, led to the remodeling of the colon mucosal barrier and its associated metabolites demonstrating its therapeutic potential in maintaining the integrity of the colon mucosa [80]. In another investigation, Zhang et al*.*, explored the anti-colitis potential of polyphenolic compounds extracted from *M. oleifera* using a DSS-induced model. Following oral administration, a noteworthy reduction in cell infiltration, inflammatory mediators, and alterations in protein expressions were observed. Additionally, in vivo assessments demonstrated a reversal of histological changes via downregulation of the NF-κB signaling pathway. All these findings highlight the promising therapeutic effects of *M. oleifera*polyphenolic compounds in mitigating colitis-related inflammation [81].

#### *Hericiumerinaceus mycelium*

*Hericium erinaceus*, commonly known as Lion's Mane and belonging to the Hericiaceae family, has demonstrated the ability to alleviate symptoms of ulcerative colitis (UC) by exerting anti-inflammatory effects. For instance, Shaoand et al. evaluated the ameliorative actions of the unique polysaccharide EP-1, isolated from *H. erinaceus* extract, against acetic acid (A.A) treated UC model of rats. Employing IlluminaMiSeq and immunoblot analysis, the study revealed noteworthy alterations in intestinal structure, an increase in fatty acids, and a significant reduction in G-protein receptors in the epithelium [82]. Similarly, Wang et al*.*, performed a research to explore the potential of the EP-1 polysaccharide against UC, utilizing Caco-2 cells in a rat model. Authors noticed that, treatment with EP-1 led to an increase in antioxidant markers, improvementsin mitochondrial function, and a reduction in apoptosis in the colon epithelium. These findings, in conjunction with the research by Shao et al*.*, further support the therapeutic potential of EP-1 in ameliorating UC. The collective evidence from both studies highlights the multifaceted benefits of EP-1, indicating its potential as a complementary herbal medicine for alleviating the symptoms and underlying mechanisms of UC [83]. The potential ameliorative effects of HEP10, an another low-weight polysaccharide derived from *H. erinaceus*, were observed in both RAW264.7 murine macrophage cells and a DSS-induced mice model. Their investigation revealed that the beneficial impact of HEP10 is due to the suppression of NLRP3 activation and the suppression of other signalling pathways. Furthermore, these mechanisms were shown to modulate the gut micro biota. This suggests that HEP10 may have a positive influence on inflammatory responses and gut health through its regulatory effects on key cellular pathways and the microbiome [84]. Durmus et al. evaluated the prophylactic efficacy of *H. erinaceus* in the context of UC by assessing microscopic and macroscopic indices in a TNBS-induced animal model. The study revealed that treatment with *H. erinaceus* resulted in a notable reduction in various markers, including NO, malondialdehyde, myeloperoxidase, and other elevated mechanisms when compared to the control group [85]. A comprehensive investigation was conducted on the anticolitic potential of an HBQ-complex, comprising *Hericium erinaceus*, berberine, quercetin, niacin, and biotin. The study focused on its effects against ex vivo inflamed colon mucosa. The findings demonstrated a consistent and progressive reduction in pro-inflammatory cytokines along with an elevation in IL-10 both at mRNA and protein levels within the inflamed tissue [86].

#### *Punica granatum*

*Punica granatum*, often referred to as pomegranate and a member of the Lythraceae family, exhibits anti-colitic properties and demonstrates promise in alleviating symptoms, potentially enhancing gut health for individuals with UC. For instance, in an investigation performed by Shah et al., the researchers assessed the potential impact of *P. granatum* juice and purified punicalagin in inhibiting NF-κBsignaling pathways using a dinitro benzoic acid-aggravated colitis model. The dinitrobenzoic acid-injected model exhibited elevated levels of proinflammatory cytokines, inflammatory mediators, neutrophil infiltrations, and decreased antioxidant markers in the colon epithelium. However, the administration of juice and punicalagin reversed these effects.Remarkably, the ameliorative effect of *P. granatum* juice was found to be more effective compared to purified punicalagin. This suggests that the combination of compounds present in the pomegranate juice may contribute to a more pronounced reversal of colitis-related markers and highlights the potential superiority of the whole juice over isolated components in mitigating inflammatory responses in the studied colitis model [87]. Kamali et al. performed a clinical trial (randomized, placebo-controlled) to investigate the effectiveness of *Punica granatum* peel extract in managing symptoms of colitis, assessed through the Lichtiger Colitis Activity Index. The results demonstrated a no table increase in the clinical response, when compared to the placebo group over the four-week study period. This suggests that the anti-inflammatory activity of *P. granatum* extract may be advantageous in the complementary management of UC [88].

#### Miscellaneous extracts

Various other extracts have also been reported in literature having profound activity against UC. For example, a pre-clinical investigation of the prophylactic potential of *Zanthoxylum alatum *seed extract for the management of UC in mice was reported by Kalyankumarraju et al. The findings of in vitro and in vivo studies using NF-κB-luciferase translocation assay and DSS-induced UC model, respectively demonstrated that hydroalcoholic extract of ZA seed could be used as a prophylactic as well as effective treatment option for stress aggravated UC [89]. Zaware et al., explored the therapeutic potential of *Mimosa pudica* extract has also been explored by assessing colonic injury biomarkers in an A.A-induced colitis model, comparing its efficacy with the standard drug prednisolone. The ethanolic extracts of *Mimosa pudica*, enriched with flavonoid derivatives, demonstrated notable efficacy in alleviating UC symptoms in rats. This was evidenced by significant inhibition of key oxidative and inflammatory mediators, including myeloperoxidase and malondialdehyde levels. The findings highlight the promising role of *Mimosa pudica *extract to regulate pathological alterations in colonic tissue [90].

Another study explored the oxidoinflammatory effects of a sicilian variety of garlic extract in an ex vivo experimental model using mouse specimens treated with lipopolysaccharide. The investigation revealed a notable reduction in inflammatory mediators, enzymes, and gene expressions induced by LPS. Both hydroalcoholic and water extracts of garlic exhibited protective effects on the colon, with the hydroalcoholic extract demonstrating a more potent anti-inflammatory effect. These findings highlight the effectiveness of garlic extracts in the management of UC, pointing towards their beneficial impact on mitigating ulcerogenic scores in the colon [91]. Obiri et al. investigated the therapeutic potential of *Cordia vignei* leaf extract in ameliorating A.A trearted UC model of rats. Histological examinations depicted a significant reduction in inflammatory scores, indicating the efficacy of this leaf extract in preventing colon tissue damage. Moreover, *C. vignei *administration significantly upregulated the antioxidant markers & suppressed serum inflammatory levels in the A.A-induced colonic environment [92]. A study was conducted to evaluate the effectiveness of *Alstonia boonei* extract in alleviating symptoms in comparison to the standard drug prednisolone, using a UC model treated with DSS. Researchers demonstrated a noteworthy inhibition of inflammatory secretions and prostaglandins, along with an elevation of antioxidant markers in colon epithelium. Histological findings further supported these outcomes, revealing an increase in body weight, which had been decreased by the induction of colitis. These collective results strongly suggest a protective role for *A. boonei* extract in inflammatory bowel disease [93]. Qin et al*.* investigated the potential mechanisms underlying the efficacy of *Rubia cordifolia L.* extract against the management of UC using a mice model treated with DSS. In vivo study revealed a dual inhibitory effect on both the formation of the NLRP3 and the activation of the JAK-STAT pathway inside the colon. This dual inhibition resulted in decrease in mortality, decreased release of inflammatory cytokines & alleviation of clinical symptoms [94]. An investigation reported the anticolitic effects of *Sesbania grandiflora* extract in a mice model of UC induced by A.A. The study demonstrated significant alterations in the DAI, restoration of biochemical parameters, and inhibition of free fatty acids in the diseased model, particularly when treated with *S. grandiflora* extract. Additionally, histological findings indicated that extract could effectively prevent cellular infiltration, necrosis, and other ulcerative signs in the colon mucosa [95]. Silva et al*.* explored the efficacy of lutein, a carotenoid extracted from *Tagetes erecta *L, for its anticolitic activity in an experimental UC model. The study revealed significant reductions in disease severity, achieved by downregulating the secretion of ulcerative biomarkers and enhancing endogenous antioxidant markers in nude mice treated with DSS. Lutein demonstrated the capacity to attenuate histological alterations, counteract oxidative stress, and increase mucin staining in the colon [96]. A preclinical investigation conducted to evaluate the potency of Ziziphusspina (ZS) extract in mitigating colon inflammation, using a rat model treated with A.A. Researchers employed HPLC and histochemical methods to analyze the effects of this extract. Findings of their study demonstrated that pretreatment of ZS fruit extract was associated with inhibition of pathological injury scores, proinflammatory cytokines & oxidative stress, alterations in body weight, mucin concentration, and apoptosis. Moreover, pre-administration with ZS extract modulated the Nrf2, HO-1 &p38 MAPK expression in A.A administered UC model, as demonstrated by quantitative Reverse Transcription Polymerase Chain Reaction (qRT-PCR) analysis. These results highlight that *Ziziphusspina* fruit extract could serve as an additive or alternative medication for the management of UC, emphasizing its potential as a therapeutic agent in combating UC-related symptoms and pathology [97]. A study explored the antioxidant defense mechanism of *Averrhoa bilimbi* fruit extract (ABFE) in comparison to the standard drug sulfasalazine, for prophylaxis against UC in A.A-treated rats. The protective effects of ABFE were proved as its administration led to significant changes in mucosal injury markers, spleen and colon tissue weight, oxido-inflammatory markers, and colon histology in treated groups compared to the control group. Findings from antioxidant & anti-inflammatory studies suggest that ABFE could serve as a potential candidate in the treatment of ulcerative colitis [98]. Chen et al. explored the protective role of *B. xiexin* decoction against a UC model treated with DSS, employing ELISA &immunohistochemistry (IHC). The findings demonstrated that *B. xiexin* decoction treatment led to decreased disease activity index, ulcer scores, levels of 8-oxoguanine, interleukins, and the results demonstrated a notable increase in the clinical response when compared to the placebo group over the four-week study period. Additionally it also inhibited the levels of (myeloperoxidase) & MDA (malondialdehyde) in the mice epithelium. Furthermore, activation of Nrf2 was observed in response to *B. xiexin* decoction treatment. These results collectively highlight the potential oxidoinflammatory properties of *B. xiexin* decoction in preventing and treating UC. The modulation of key markers and pathways associated with inflammation and oxidative stress suggests *B. xiexin* decoction as a potential candidate for therapeutic intervention in treating UC [99]. Several medicinal plants have also been explored. For instance, Lie et al*.*, have demonstrated anti-ulcerative potential of *Saussurea Pulchella* and their research involved analyzing the chemical composition of the extract, studying its effects on UC symptoms in mice (Adult male BALB/c mice). The findings revealed that the extract reduced UC symptoms, inflammation, and oxidative stress while improving colon health. Key metabolites, biological targets, and metabolisms related to extract effectiveness against UC were identified. Overall, the study suggests that this extract could serve as a promising natural treatment for UC [100]. In another study, Rehman et al*.*, aimed to explore the potential of *Calliandra haematocephala* in treating UC by assessing its impact on inflammatory mediators and oxidative stress markers using rat models. Results showed that both extracts mitigated inflammation, reduced colon ulceration, normalized oxidative stress markers, and modulated gene expression related to inflammation [101]. Devi et al*.*, investigated role of *Bacopa monnieri* in management of A.A.-induced UC in mice. Acetic acid infusion caused severe colon inflammation and increased myeloperoxidase activity. Treatment with *B. monnieri* extract and its saponin-rich fraction significantly suppressed inflammation, myeloperoxidase levels, and disease activity score in a dose-dependent manner [102]. Jia et al*.*, have evaluated the potential of ethanolic extract of *Limonium bicolor* (a traditional Chinese medicine) in both cell and mouse models of UC. Outcomes showed that the extract suppressed cytokine secretion in macrophages and exhibited protective effects against UC in mice, reducing disease severity and restoring colon health. Additionally, it reversed microbial imbalances associated with UC. Network pharmacology analysis identified key compounds in extract linked to inflammatory genes, enhancing understanding of its therapeutic potential and paving the way for UC treatment development [103]. Abdellatif et al. aimed to evaluate the total phenolic and flavonoid contents, antioxidant properties, chemical composition, and anticoloitis potential of *Cassia fistula* leaf extract in A.A induced UC in male rats. The extract demonstrated dose-dependent efficacy in treating UC in rats, improving hematological parameters, liver biomarkers, oxidative stress, and histopathological features. Molecular docking studies revealed the potential anti-inflammatory and antiapoptotic activities of *C. fistula* compounds, particularly emodin and isorhamnetin, which exhibited strong binding affinities to COX-2 and caspase-3 proteins, respectively [104]. Cumulative results of all findings highlight the effectiveness of various extracts in the management of UC and this could act as a potential candidate for management of UC.

### Essential oils

Several preclinical studies demonstrated that essential oils can enhance the equilibrium of GIT immunity through their ‘anti-inflammatory activity’ and the downregulation of pro-inflammatory mediators (Table 4) [105]. Also essential oils can lead to suppression or activation of various protein expressions and translocation of signaling pathways and thus mitigating UC [106].

#### Carvacol

An investigation is carried out using a colitis model induced with A.A to evaluate the antioxidant and anti-inflammatory efficacy of a phenolic monoterpene, carvacol revealed that pretreatment with carvacol showed decreased level of inflammatory mediators, DAI, macroscopic and microscopic damages and also significant elevation of antioxidant enzymes in the colon tissue [107]. Another investigation demonstrated the anticolitic effect of carvacol is associated with the protection of mucosal barrier through reducing translocation of NF-κB pathway and inhibiting TLR4 receptor activation pathway in DSS induced colitis model. It also ameliorates UC in the colon by inhibiting inflammatory scores and cell apoptosis caused by induction of LPS (Liu et al. 2022). The results of this study highlight that, carvacol could be a prophylactic intervention in the management of UC [108].

#### d-Limonene

An in vivo analysis demonstrated the ‘anticolitic’ effects of d-limonene for the prevention of UC against a TNBS administered colitis model. The induction of colitis with TNBS caused elevated levels of pathological scores via activation of NF-κB translocation in the colon epithelium which has been reversed by treatment with d-limonene [109]. Lihua et al*.* 2016 demonstrated the potential oxidoinflammatory activity of d-limonene for the management of UC using rats treated with DSS. Their findings indicated a notable suppression of matrix metalloproteinase, TGF-β expression and increase in antioxidant markers in limonene treated rats, suggesting the protective role of d-limonene in the management of UC [110]. Another in vivo study was carried out by Estrella et al. to investigate the ameliorative action of d-limone in oxazolone treated colitis model. Prevention of mucosal damage, hyperalgesia, pathological biomarkers and major antinociceptive activity was observed in limonene treated rats. The cumulative results from above mentioned studies demonstrated the curative potential of d-limonene for the prophylaxis of UC [111].

#### Thymol

A colitis model treated with DSS was employed to investigate the mechanism and protective efficacy of thymol in treating UC. Thymol treatment demonstrates a beneficial impact on experimental colitis by effectively reducing pathological scores, mitigating mucosal damage, and suppressing pro-inflammatory cytokines. Additionally, it elevates antioxidant markers via inhibition of nuclear factor pathway activation in mice [112]. Another study explored the anticolitic activity of thymol against the management of colitis, using a rat model treated with A.A. The colon mucosa of rats showed marked inhibition of histological damages, pro-inflammatory cytokines and elevation of protein (pNFκB p65) via down regulation of NF-κB pathway [113]. The more study demonstrated the effectiveness of thymol in same colitis model by comparing it with a reference compound (prednisolone). Treatment with thymol exhibited better anticolitic effect as compared to prednisolone group by reducing all pathological scores in the rat colon cells. The above mechanism depicts the potential activity of thymol in the prophylaxis of UC [114].

#### Menthol

The oxidoinflammatory efficacy of menthol evaluated in A.A treated rats for the management of UC revealed that pre-treatment with menthol significantly inhibited mucosal damage, inflammatory levels and histopathogical scores in the colon cells at medium to higher dose as compared to a standard drug dexamethasone [115]. Another study has demonstrated the anticolitic activity of the menthol by using A.A induced model in rats. Due to A.A administration, a significant reduction in ulcer scores, antioxidant markers, glutathione levels and also a raised level of malondialdehyde, lipid peroxidation activity were observed in rats. Treatment with menthol attenuates all the above ulcerogenic scores in the colon mucosa [116]. Jing et al. reported the ameliorating efficacy of menthol for the treatment of colitis-associated caner by using AOM-DSS/AD (azoxymethane combined with dextran sulphate sodium) mouse model. Administration of menthol to the mouse causes significant reduction of histopathological scores, DAI, proliferation biomarkers and inflammatory mediators in the colon epithelium. In addition menthol supplement diet also improves the tumorigenesis, growth of butyrate producing bacteria in AD induced mouse model [117]. The above reports depicted that menthol may be an advantageous therapeutic target in the management and prevention of UC.

#### 1,8-cineole (Eucalyptol)

An in vitro and histopathological study was conducted, to evaluate the targets and ‘anti-inflammatory’ mechanisms of 1, 8-cineole (Eucalyptol) by using UC model treated with DSS. In vitro studies revealed, 1, 8-cineole (Eucalyptol) inhibits the macrophage polarization by affecting the formation of HSP90- SGT1-NLRP3 complex. Authors have also evaluated that 1, 8-cineole (Eucalyptol) also improved the epithelial barrier disruption and pathological symptoms in DSS induced mice [118]. Venkataraman et al. conducted a molecular docking analysis to assess the agonistic activity of eucalyptol towards the PPARγ protein, employing both in vitro (TNF-α-stimulated HT-29 cells) and in vivo approaches (DSS-induced colitis model). Authors reported that, eucalyptol could exert potent anticolitic activity by activating PPARγ protein and Nrf2 translocation inside the epithelium [119]. In an another study Sandeep et al. have demonstrated the anticolitic effect of 1, 8-cineole (Eucalyptol) using LPS-stimulated RAW macrophages and DSS induced experimental model. Pretreatment with 1, 8-cineole (Eucalyptol) demonstrated a dose responsive inhibition of DAI; pro inflammatory cytokines and other pathological symptoms in DSS treated mice. Furthermore, it alleviated ulcer scores in the colon epithelium by suppressing the expression of NF-κB and COX-2 in both the experimental models [120]. The above findings suggested that 1, 8-cineole could be a valuable candidate for the management of UC.

### Phytocompounds

Medicinal plants contain numerous chemical compounds (phytocompounds), which are known for their promising therapeutic effect in colon inflammation and are used since years [121, 122]. Several researchers have evaluated the potential of these phytocompounds (Table 3) and underlying anti ulcerative mechanisms using various animal models that have been summarized below:

**Table 3:** Anticolitic potential of Essential oil and Phytocompounds

Sl.No	Essential oils/phytochemicals	Source	Mechanism of ulcer healing	In vitro*/*In vivo models used	Dose/concentration	References
1	Carvacol	*Origanum onites L*	Decreased abdominal hyperalgesia Inhibited lipid peroxidation Suppressed secretion of inflammatory biomarkers	A.A treated UC model of mice	25, 50 or 100 mg/kg	[107]
		*Origanum onites L*	Suppressed TLR4 pathway activation Deactivated NF-κB translocation Downregulated cleaved-caspase 3 protein levels	LPS induced colonic epithelial cells and DSS treated UC model of rat	50 and 100 mg/kg	[108]
**2**	d-Limonene	Oils of orange, grapefruit and lemon	Inhibited NF-κB translocation Suppressed secretion of inflammatory cytokines	HT-29/B6 cells and TNBS treated UC model	10 mg/kg	[109]
		oils of orange, grapefruit and lemon	Reduced matrix metalloproteinase gene and COX-2 expressions Possessed anti-oxidant and anti-inflammatory activity	TNBS treated UC model of rat	50 or 100 mg/kg	[110]
		*Agastache mexicana*	Possessed antinociceptive efficacy Reduced hyperalgesia, pathologicalscores, and mucosal inflammatory cytokines	Oxazolone treated UC model	3–300 mg/kg	[111]
3	Thymol	*Thymus vulgaris*	Suppressed expressions viz. of TNF-alpha, IL-1β etc. in the colon Suppressed the activation of NF-κB inflammatory pathway	DSS treated UC model of mice	60 mg/kg	[112]
		*Thymus vulgaris*	Downegulated the levels of NF-κB p65 protein expression Decreased the production of inflammatory mediators	A.A treated UC model of rat	10, 30, and 100 mg/kg per day	[113]
		*Thymus vulgaris*	Reduced COX-2 expression Inhibits NF-κBp65 translocation Suppressed myeloperoxidase activity, NO level and malondialdehyde intensity	A.A treated UC model of rat	100 mg/kg	[114]
4	Menthol	*Mentha canadensis*, *Mentha pipereta*	Reduced serum inflammatory secretions Ameliorated pathological alterations and improved hematocrit in colon tissues	A.A treated UC model of rats	20, 50 and 80 mg/kg	[115]
		*Mentha canadensis*, *Mentha pipereta*	Reduced the ulcer scores raised by acetic acid Suppressed the secretion of inflammatory cytokines Elevated anti-oxidant markers	A.A treated UC model of rats	50 mg/kg/day	[116]
		*Mentha canadensis*, *Mentha pipereta*	Down regulated the histopathological scores and lowers the expression of proliferation biomarkers Ameliorated gut microbiota dysbiosis	DSS treated tumorigenesis model	1, 1.5 and 2 g/100 g	[117]
5	1, 8-cineole (Eucalyptol)	*Amomum compactum sol*	Inhibited the macrophage M1 polarization Reduced ATPase activity and NLRP3 inflammasome activation	LPS-induced RAW264.7 cells and DSS treated UC model of mouse	50, 100, and 200 μM	[118]
		*Amomum compactum sol*	Increased PPARγ activity Suppressed the inflammatory secretion in colon tissue Increased translocation of Nrf2 pathway	TNFα-stimulated HT-29 cells and DSS induced colitis model	50, 100 and 200 mg/kg.body weight/day	[119]
		*Amomum compactum sol*	Activated PPAR-γ protein expression Suppressed NF-κB pathway Reduced pro-inflammatory cytokine secretion	LPS-induced RAW 264.7 macrophages and DSS treated UC model of mice	100 & 200 mg/kg body	[120]
6	Corynoline	*Corydalis bungeana Turcz*	Promoted Nrf2 nuclear activation Suppressed NF-κB pathway and reduced phosphorylation Down-regulated pro-inflammatory mediator levels	DSS treated UC model of mice	NA	[124]
7	Coptisine	*Coptis chinesis*	Inhibited macrophage M1 polarization Enhanced CCAAT enhancer binding protein Increased the levels of METTL 14 Reduced the expression of MAPK	DSS treated UC model of mice	NA	[125]
8	Cepharanthine	*Sephania cepharantha*	Inhibited Aconitate decarboxylase 1expression Reduced macrophage infiltration and secretion of inflammatory cytokines Modulated gut micro biota dysbiosis	LPS-induced RAW264.7 macrophages and DSS treated UC model of mouse	NA	[126]
9	Protopine	*Macleaya cordata*	Suppressed the inflammatory genes expressions Restored epithelial mucin secretion and modulated tight junction proteins expressions Improved gut homeostasis	DSS treated UC model of mice	NA	[127]
10	Oxysophocarpine	*Sophora alopecuroides*	Reduced the phosphorylation of nuclear factor-κB Modulated oxidative stress Ameliorated cell destruction and cell infiltration	DSS treated UC model of mice	NA	[128]
11	Coumaric acid and Syringic acid	Fruits and vegetables	Elevated the levels of HO-1, Nrf2 and mRNA Decreased the release of proinflammatory cytokines Increased antioxidant defense system	A.A treated UC in rats	Coumaric acid (150 mg/kg), Syringic acid (50 mg/kg)	[129]
12	Wedelolactone	*Wedelia calendulace* and *Eclipta alba*	Suppressed the IL-6/STAT3 inflammatory pathway Inhibited inflammatory secretions &pathological alterations in colon tissue	DSS treated UC model of rat	100 mg/kg/day,	[130]
13	Puerarin	*Radix Puerariae*	Restored the gut microbiota composition and dysbiosis Alleviated colonic tissue morphology and inflammatory factor expression	DSS treated UC model of mice	200 mg/kg Body weight	[131]
14	Galangin	*Alpinia officinarum Hance*	Inhibited NLRP3 inflammasome activation Regulated fatty acid synthesis Reduced HSP90β expression	DSS induced mice model of colitis	40 mg/kg	[132]
15	Cardamonin	*Alpinia katsumadai*	Inhibited necroinflammation Blocked the phosphorylation of necrosome containing receptor-interacting protein kinase	LPS plus SZ (LSZ)-stimulated HT29, L929, or RAW264.7 cell and DSS treated UC model	10 mg/kg/day	[133]
16	Genistein	Soybeans and fava beans	Enhanced mitochondrial biogenesis Inhibited cell apoptosis Elevated the Nrf2, HO-1 expressions	Acetic acid induced colitis model of rat	25 mg/kg	[134]
17	*N*-acetyl cysteine	*Allium cepa*	Clinical study	NA	800 mg	[136]
18	Diallyl Trisulfide	*Allium sativum*	Modulated phosphorylation of focal adhesion kinase Accelerated the endocytosis of integrin β1 Promoted mucosal repair	DSS treated UC model of mice	10 μmol/day	[137]
19	6-(methylsulfinyl) hexyl isothiocyanate (6-MITC)	*Wasabia japonica*	Inhibited GSK-3β and NF-κB pathways Alleviated colonic alterations Decreased secretion of inflammatory mediators	DSS treated UC model of mouse	NA	[138]
20	Sulforaphane	Broccoli, cabbage, kale, and arugula	Regulated gut microbiota dysbiosis Activated Nrf2 signaling pathway Inhibited macrophage polarization	DSS treated UC model of mice	10, 20, 40 mg/kg	[139]
21	Ergothionene	*Pleurotus ostreatus*	Inhibited TLR4/MyD88/NF‐κB pathway Inhibited the secretion of proinflammatory secretions Ameliorated pathological alterations	DSS treated UC model of rat	2 mg/kg body weight	[140]

#### Nitrogen containing compounds

Nitrogen containing secondary metabolites, especially derived from medicinal plants exhibits a potent anti-inflammatory and immune regulating properties against UC [123]. For instance Zhang et al. considered an alkaloidal compound *Corynoline*, extracted from *Corydalis bungeana Turcz*, to evaluate its ‘anti-inflammatory’ activity against UC mice treated with DSS. Their findings demonstrated, *corynoline* treatment reduced all the ulcerogenic markers in DSS administered coitic mice and also down-regulated the inflammatory cytokines, oxidative stress markers in the intestinal tissue by restraining the activated NF-κB translocation [124]. Another study evaluated the mechanism and curative effect of *coptisine*, using DSS treated mice for the treatment of UC. The authors demonstrated, *coptisine* exhibits an inhibitory effect on macrophage polarization by regulating m6A methylation of tuberous sclerosis complex. In addition, *coptisine* treatment ameliorated the colitic symptoms and inhibited the inflamed lesions in mice administered with DSS [125]. Zhang et al. investigated the immunomodulatory action of Cepharanthine by conducting both in vitro experiments on LPS-administered RAW264.7 macrophages and in vivo experiments on a colitis model administered with DSS. Their analysis demonstrated that, *Cepharanthine* inhibited the Aconitate decarboxylase 1 over-expression, macrophage cell infiltration and also maintains the gut microbiota homeostasis to combat UC [126]. Anti inflammatory activity of an isoquinoline alkaloid, *protropine* was evaluated by Yue et al*.* using DSS administered colitis model. The study demonstrated that, *protropine* administration effectively ameliorated UC by maintaining gut microbiota dysbiosis, inhibiting inflammatory gene expressions and modulating intestinal mucosal barrier integrity significantly [127]. In another investigation, Liu et al. evaluated the mechanism and impact of *Oxysophocarpine* on UC by using DSS treated mouse model. *Oxysophocarpine* administration alleviated colitis by suppressing the level of phosphorylation of nuclear factor-κB and theTNF receptor-associated Factor 6 (TRAF6) in DSS treated mouse. Moreover, *Oxysophocarpine* reduced DAI, inflammatory cell infiltration, colon injury and oxidative stress in the colon tissue [128].

#### Phenol containing compounds

Polyphenolics or flavanoids are important among all the secondary metabolites that possess a strong ‘anti-oxidant and anti-inflammatory’ properties and also been reported to have efficacious against UC [17]. For instance, Mahsa et al*.* used a colitis model induced with A.A, to investigate the anticolitic efficacy of two phenolic derivatives i.e. *coumaric acid* and *syringic acid*. A notable suppression of inflammatory cytokines and upegulation of NQO1 mRNA, HO-1, and Nrf2 expression was observed in the epithelial colonic tissue, as compared to the A.A group [129]. Prakash et al. have investigated in vivo anticolitic effects of *wedelolactone* using DSS administered UC model of female albino wistar rats by targeting interleukin-6/signal transducer pathway. From ‘anti-inflammatory’ study, it was demonstrated that, pro inflammatory cytokines expression was reduced significantly and blockage of IL-6/STAT3 pathway preserved after exposure of *wedelolactone* [130]. An in vivo investigation in C57BL/6J mice was performed to evaluate the anti-inflammatory effects of *Puerarin* in colitis. Researchers observed that *Puenarin* treatment alleviated the crypt deformation, inflammatory cell infiltration and reduced the pro-inflammatory cytokine markers in the colon mucosa of mice and well act as a potent therapeutic agent that could be used in UC for maintaining gut microbiota homeostasis [131]. A natural flavonoid *Galangin* has been demonstrated to have protective effect on UC in an in vivo animal model. Molecular biology techniques demonstrated that, *galangin* can ameliorate colitis by inhibiting HSP90β oligomerization and restraining inflammasome activation in the colon tissue [132]. The anticolitic effect of *Cardamonin *via necroptosis inhibition by using lipopolysaccharide administered HT29, L929, or RAW264.7 cell have been reported. Shen et al. have reported that, *Cardamonin* prevents the necrosome formation by blocking RIPK1/3 phosphorylation in the colon tissues. Furthermore, oral treatment of *Cardamonin* alleviated the colitis symptoms, restrains the colon damage and inhibits necroinflammation in DSS administered mice suggesting that *Cardamonin* could serves as a novel inhibitor of necroptosis and holds promise as a potential candidate for treating UC [133]. Another study investigated the therapeutic effects of another phenolic compound i.e. *Genistein* against UC by using rat model treated with A.A. Administration with a dose of 25-mg/kg, *genistein* ameliorated the pathological symptoms, colon damage and cell infiltration in A.A injected rats. A remarkable balance of gene and protein expressions has been observed in the colon tissue. Moreover, antioxidant potential of this phenolic compound increases mitochondrial biogenesis followed by reducing cell apoptosis, suggesting its beneficial role [134].

#### Sulphur containing compounds

Sulphur containing compounds isolated from natural sources has been verified for its excellent anti-inflammatory activity in various preclinical models against the treatment of UC. They exert their anti-inflammatory action by inhibiting several signaling pathways [135]. For instance, anti-inflammatory potential of *N-acetylcysteine*has been investigated on remission maintenance in 168 UC patients in a clinical trial (double blind randomized control) via grouping them as NAC (*N*-acetylcysteine) group & placebo group. Findings of this study revealed a significant reduction of remission in NAC group as compared to the placebo group [136]. In another investigation, Zhang et al. evaluated the colonic mucosal healing effect of *diallyltrisulfide* in mice after induction of colitis using DSS. Their findings demonstrated that diallyltrisulfide improved cell migration, wound healing, focal adhesion kinase phosphorylation in the intestinal epithelial cells by promoting endocytosis of integrin β1 and thereby promoting mucosal healing [137]. Lohning et al*.* used a chemically induced murine model and performed in silico techniques, to investigate the pathways by which 6-MITC (6- (methylsulfinyl)hexyl isothiocyanate) restrains inflammation in the epithelial cells. It was observed, 6-MITC treatment ameliorates the intestinal inflammation by suppressing NF-kB signaling through GSK-3b (glycogen synthase kinase 3 beta) inhibition and also improves colonic alterations in DSS induced mouse [138]. Similarly, Zhang et al. explored the anti-inflammatory activity of *sulforaphane* in UC mice treated with DSS. After DSS induction, the mice showed, decreased body weight and increased colon damage, intestinal dysbiosis, myeloperoxidase activity in the colon tissues. Meanwhile, administration of sulforaphane reversed all the pathological symptoms and modulated the gut homeostasis in DSS treated mice [139]. The protective role of a sulphur containing aminoacid, *ergothionene* against UC was studied in DSS treated animal model. Authors reported that *ergothionene* alleviates colon damage; pathological symptoms in DSS induced rats. Furthermore, it inhibited the expression of TLR4/MyD88/NF-κB pathways and exhibited potent antioxidant activity. [140].

### Nutraceuticals

Nutraceuticals play a vital role in treating UC via modulating gut microbiota composition, regulating immune system and maintenance of remission [141, 142]. Enormous studies have been reported where nutraceuticals (Table 4) such as vitamins, carbohydrates, bioactive polypeptides, prebiotics, probiotics and natural oils have exhibited potential preventive effect in UC as summarized below:

**Table 4:** The protective effect of nutraceuticals in the management of ulcerative colitis

Sl. No	Nutraceutical name	Mechanism of ulcer healing	In vitro/In vivo models used	Dose	References
*Vitamins*					
1	Vitamin A	Decreased Mayo clinic score Attenuated clinical remission, response and pathological scores Maintained the balance between Th17 and Treg cells	Clinical study	25000 IU/day	[143]
2	Vitamin C	Reduced the levels of fibroblasts & collagen types	DSS treated UC model of mice	4 g/kg	[144]
3	Vit C with Vit D3	Decreased the Notch-1 protein expression Claudin-2 expression was reduced	LPS-induced SW480 cells and DSS treated UC model of guinea pig	200 IU/kg/day Vit-D3 + 100 mg/ kg d Vit-C	[145]
4	Vitamin D	Reduced the secretion of inflammatory mediators Attenuated Mayo score and intestinal barrier function	NA	NA	[146]
5	Vitamin E	Maintained the levels of tight junction compositions Modulated gut microbiota dysbiosis	DSS treated UC model of mice	300 mg daily intake for a 60 kg adult	[147]
*Carbohydrates*					
6	Dandelion root polysaccharides	Triggered Nrf2 activation Attenuated ulcerogenic scores Modulated gut mucosal homeostasis	DSS treated UC model of C57BL/6 mice	150 or 300 mg/kg/day	[149]
7	Atractylodes macrocephala	Restored gut microbiota dysbiosis Modulated the formation of short-chain fatty acids and metabolism of gut microbiota composition	DSS treated UC model of mice	10, 20, 40 mg/kg bw	[150]
8	*Morinda citrifoli*	Inhibited COX-2 expression Suppressed the levels of pro-inflammatory cytokines and oter inflammatory mediators	A.A treated UC model of mice	0.1, 0.3, and 3.0 mg/kg	[151]
9	*Rheum tanguticum*	Regulated gene expression associated with Notch and NF-κB pathways Down-regulated the oxidoinflammatory markers	DSS treated UC model of mice	NA	[152]
*Prebiotics*					
10	*Lactobacillus acidophilus* PIN7	Down-regulated the Th2 cell‐mediated proinflammatory secretions Elevated the anti inflammatory NLRP6 &IL‐18 levels Suppressed COX-2 enzyme expression	DSS treated UC model	NA	[153]
11	*Bifidobacterium longum*	Inhibited NF-κB immunoinflammatory pathway Suppressed the release of proinflammatory cytokines	DSS treated UC model	NA	[154]
*Probiotics*					
12	Alginate oligosaccharides	Up-regulated the expressions of Bcl-2 proteins and E-cadherin Modulated the gut microbiota contents	DSS treated UC model	200 mg / kg body weight	[156]
13	Fructooligosaccharides-SCFA esters	Restored intestinal homeostasis Maintained gut microbiota dysbiosis	Human colonic fermentation model	NA	[157]
*Bioactive polypeptideds*					
14	Lupin protein	Reduced MMP-9 activity Regulated oxidative stress and inflammatory secretions	TNBS treated UC model of mice	0.1 to 10 g/kg	[158]
15	Foxtail millet protein hydrolysates	Inhibited NLRP3/ASC/caspase-1 pathway Suppressed inflammasome over expression and IL-1β levels	LPS induced Caco-2 cells and DSS treated UC model of mice	NA	[159]
16	Tuna bioactive peptide	Increased short chain fatty acid levels Elevated mucosal barrier protein levels Reduced the secretion of inflammatory mediators	DSS treated UC model of mice	200 mg/kg and 500 mg/kg	[160]
*Natural oils*					
17	Walnut oil	Modulated protein expressions of tight junctions, free fatty acid receptors Reduced release of proinflammatory mediators	DSS treated UC model of mouse	2.5 ml/kg/day	[162]
18	Olive oil	Suppressed the STAT3, pSTAT3, COX-2 pathways expression Decreased apoptosis Attenuated cell proliferation	DSS treated UC model of rat	125 ± 25 mg/kg	[163]
19	Flaxseed oil	Regulated oxidative condition Restored microbial alterations	DSS treated UC model of rats	400, 800 and 1600 mg/kg body weight	[164]
20	Cottonseed oil	Decreased pro-inflammatory cytokine secretion Reduced expression of oxidative stress markers	DSS treated UC model of mouse	NA	[165]

#### Vitamins

A randomized controlled clinical trial to calculate DAI was done by using mayo clinic score on 150 patients. Results demonstrated that intake of Vit-A at a dose of 25000 IU/day could improve mucosal damage and disease activity index [143]. A study was carried out in DSS administered mouse, to examine the ameliorative action of Vit-C against UC. The elevated levels of inflammatory cytokines and reduction in fibroblast, collagen expressions in the colon cells were reversed by treatment of high dose Vit-C in DSS induced mouse [144]. Another study has demonstrated that, combination of Vit-C with Vit-D3 could attenuate intestinal barrier, tight junction proteins by regulating Notch-1 signaling pathway in DSS induced guinea pig model facilitating treatment of UC [145]. Guo et al. conducted a meta analysis to evaluate clinical safety and effectiveness of Vit-D in treating UC by using RevMan 5.4 software. Results demonstrated that treatment with Vit-D was more effective than conventional treatment groups in ameliorating mayo score and epithelial barrier function, and downregulating inflammatory mediators in the patients [146]. The protective effect of alpha- and gamma-tocopherol against a DSS induced colitis model was also observed. The treatment with tocopherol attenuated the symptoms caused by DSS administration and also modulated gut microbiota [147].

#### Carbohydrates

Carbohydrates have also demonstrated ameliorative effect in UC [148]. For instance, Yan et al. carried out both in vitro and in vivo analysis to explore the potential effectiveness of dandelion polysaccharides (DP) in IEC-6 cells by using UC model treated with DSS. In vitro findings highlighted the efficacy of DP in suppressing inflammation, ferroptosis, and oxidative stress via activation of Nrf2pathway. Additionally, DP maintained intestinal permeability and restored mitochondrial damage by enhancing iron transport. Moreover, it ameliorates the DAI and inflammatory scores in mice treated with DSS [149]. Polysaccharides from *Atractylodes macrocephala* (*AMP) *also regulated the gut homeostasis by inhibiting the production of short-chain fatty acids and bile acids as demonstrated by Feng et al. Administration of AMP significantly reduced the mucosal injury and histopathological scores in DSS treated colitis model [150]. The anti inflammatory activity of polysaccharides (PLS), extracted from *Morindacitrifoli*has also been observed in a UC model induced with A.A. Treatment with PLS was demonstrated to reduce oxidative stress, lower inflammatory scores, and decrease cox-2 expression, suggesting its promising potential in treating UC [151]. A recent research has demonstrated the anticolitic effect of polysaccharides from *Rheum tanguticum* via modulating gut microbiota dysbiosis in a mice model induced with DSS. The heteropolysaccharide present in RTP improved the pathological symptoms, reduced oxidative stress, proinflammatory cytokines and modulates the gene expressions related to NF-κB signaling pathway in DSS treated mice [152].

#### Probiotics and prebiotics

An investigation aimed to evaluate the mechanism of Lactobacillus acidophilusPIN7 supplement diet in modulating the gut microbiota employing DSS aggravated colitis model. The PIN7 groups treated with lysozyme and heat-killed variants demonstrated elevated colonic expressions of toll-like receptors and epithelial junction proteins, along with reduced expressions of pro-inflammatory mediators, p-IκBα and mucosal injury scores in the colons of colitis mice [153]. Zhang et al. evaluated the anticolitic efficacy of *Bifidobacterium* strains (*B. longum* FBJ20M1, *B. longum* FGDLZ8M1, *B. longum* FGSZY16M3, and B. longum FJSWXJ2M1) by using DSS induced colitis model. All strains were found to be effective in ameliorating disease scores, colon injury and epithelial permeability and the *B. longum* FGDLZ8M1 strain exhibited a greater efficacy as compared to other three strains [154].

Various prebiotics have also show beneficial effects against UC [155]. In a recent study, the anticolitic potential of alginate oligosaccharides was observed i.e. alleviation of DAI and intestinal barrier damage in a DSS induced model. The alginate oligosaccharides relieve UC by modulating intestinal homeostasis, reducing Bax protein expressions and elevating Bcl-2 protein expressions in colon mucosa [156]. In the year 2023 Chen et al. performed an in vitro study to investigate the modulating effects of fructooligosaccharides-short chain fatty acid (FOS) esters i.e. butyrylated FOS and propionylated FOS in UC patients by assessing the short-chain fatty acid levels during fermentation. Both esters demonstrated efficacy in modulating intestinal homoeostasis and inflammation [157].

#### Bioactive polypeptides

Mota et al*.* used a functional food lupin protein concentrate, to evaluate its anicolitic efficacy against TNBS and A.A induced ulcer model. An oligomer, deflamin present in lupin seeds have demonstrated to be efficacious in ameliorating the pathological symptoms of UC in both the models by inhibiting MMP-9 inflammatory pathway [158]. Another bioactive peptide, foxtail millet protein hydrolysates (FMPH) also reduced inflammatory markers in DSS aggravated UC mice by inhibiting DAI, inflammasome activation, NF-κB phosphorylation through NLRP3/ASC/caspase-1 pathway [159]. Tuna bioactive peptide also depicts anti-inflammatory activity against UC model treated with DSS. Administration of tuna bioactive peptide, significantly improves the morphological and ulcerogenic scores in UC mice. It acts by increasing antioxidant markers, tight junction proteins and short-chain fatty acid levels [160].

#### Natural oils

Numerous studies have demonstrated that incorporating natural oils into the diet or using them topically could potentially have therapeutic benefits for UC, as they can modulate the gut microbiota, inhibit inflammation and improve intestinal homeostasis [161]. For instance, Bartoszek et al. performed an in vivo study to investigate the anticolitic effect of walnut oil in mouse. The presence of linoleic acid and linolenic acids in the walnut oil suppress the inflammatory cytokines and improves colon damage, intestinal permeability by restoring tight junction proteins [162]. The anticolitis effect of extravirgin olive oil (EVOO) has also been observed in DSS induced rats. The study evaluated the ameliorative effect of this oil by examining DAI, histological scores and biochemical markers in the colon of rats and found promising outcomes [163]. The administration of flaxseed oil, obtained from *Linum usitatissimum *Lin DSS induced rats reversed the ulcerogenic scores such as lowered the DAI and reduced the oxidative stress [164]. Another study using cottonseed oil demonstrated the anticolitic activity of this oil by using DSS aggravated colitis model. The animal model showed significant decrease in DAI, biochemical markers, inflammatory scores including 8-hydroxyguanosine and nitrotyrosine in colon mucosa which were aggravated by DSS administration. In addition, treatment with cotton seed oil reduced alpha-smooth muscle actin and type I collagen in order to inhibit intestinal fibrosis in the DSS induced model [165]. The above studies provide beneficial therapeutic effects of natural oils in the prevention of UC.

## Clinical research

While numerous herbal treatments for UC are explored, their safety for human use must be ensured. In recent decade, clinical trials in UC have evolved (Table 1). For instance, in a double-blind clinical trial study Uchiyama et al*.*, evaluated the efficacy of *Indigo naturalis*, extracted from Assam indigo. In their study, 46 UC patients received either *Indigo naturalis* or placebo for 2 weeks. Results showed significant improvement in the patients receiving *Indigo naturalis* compared to the placebo group. Short-term *Indigo naturalis* use appears effective and safe for UC treatment [166]. Papada et al*.*, conducted a Randomised, Double-Blind, Placebo-Controlled Trial to examine the impact of *Pistacia lentiscus*supplement on oxidative stress markers and plasma-free amino acids in active IBD patients. *Pistacia lentiscus* supplementation led to significant reductions in LDL levels and ratios in the intervention group, suggesting its potential as a non-pharmacological intervention to mitigate oxidative stress in IBD. Additionally, *Pistacia lentiscus* supplementation ameliorated decreases in plasma-free amino acids observed in patients with UC receiving placebo, indicating a potential role in improving metabolic profiles in UC [167]. Similar trial was performed by Baghizadeh and his co-authors to investigate the efficacy of *Plantago major* seed supplementation on UC symptoms. Sixty-one subjects received either 3600 mg/day of roasted *Plantago major* seed or roasted wheat flour for 8 weeks alongside standard medications. The Lichtiger Colitis Activity Index was used to assess variables at baseline, week 4, and week 8. Results showed that abdominal tenderness, gastroesophageal reflux, gastric pain, visible blood in stool, distension, and anal pain were significantly reduced in the *Plantago major* group compared to the placebo group [168].

## Conclusion and future perspectives

The chronic, relapsing nature of UC and the rising global prevalence of UC, especially in developed nations, necessitate the urgent need for effective therapeutic strategies. The present review has highlighted the significance of natural compounds, including plant extracts, essential oils, nutraceuticals, and phytochemicals, in offering protective, therapeutic, preventive, and ameliorative effects on colonic inflammation. The diverse array of natural compounds reviewed in this article, with their oxidoinflammatory properties, represents a promising avenue for the management of UC. These compounds not only provide relief to patients but also offer potential advantages in terms of reduced toxic side effects, contributing to enhanced overall well-being. Evidence supporting the efficacy of natural substances encourages further research, particularly rigorous clinical trials and translational studies, to validate their effectiveness across diverse patient populations. A deeper understanding of their underlying mechanisms is crucial for optimizing treatment strategies. Personalized medicine approaches, which consider genetic and environmental factors, may enhance the precision and effectiveness of natural compound-based interventions.Moreover, ongoing research into the intricate interplay between the autoimmunity, gut microbiota, and environmental factors in the context of UC will provide valuable insights.Future research should focus on the interplay between autoimmunity, gut microbiota, and environmental factors in UC. This knowledge will guide the development of novel prophylactic interventions that leverage the synergistic effects of natural compounds, targeting specific pathways involved in UC pathogenesis. Furthermore, exploring novel delivery systems, such as nanoparticles or targeted drug delivery techniques, could improve the bioavailability and therapeutic potential of natural compounds, enhancing their efficacy in UC treatment.Overall, integrating natural compounds into comprehensive treatment approaches holds promise for revolutionizing the management of UC and warrants continued investigation.

## Data Availability

Authors agree to share the data whenever required.

## Abbreviations

A.A: Acetic acid
DAI: Disease activity index
DSS: Dextran sulphate sodium
IBD: Inflammatory bowel disease
JAK-STAT: Janus kinase/signal transducers and activators of transcription
NF-κB: Nuclear factor kappa B
Nrf2: Nuclear factor erythroid 2–related factor 2
NLRP3: Nucleotide-binding domain, leucine-rich–containing family, pyrin domain–containing protein-3
NO: Nitric oxide synthase
PPAR-γ: Peroxisome proliferator-activated receptor gamma
TLR: Toll like receptor
TNBS: 2, 4, 6-Trinitrobenzene sulfonic acid
UC: Ulcerative colitis

## References

[1] Buie MJ, Quan J, Windsor JW, Coward S, Hansen TM, King JA, Kotze PG, Gearry RB, Ng SC, Mak JW, Abreu MT. Global hospitalization trends for Crohn’s disease and ulcerative colitis in the 21st century: a systematic review with temporal analyses. Clin Gastroenterol Hepatol. 2023;21(9):2211–21. 10.1016/j.cgh.2022.06.030. 35863682 10.1016/j.cgh.2022.06.030

[2] Wei SC, Sollano J, Hui YT, Yu W, Santos Estrella PV, Llamado LJ, Koram N. Epidemiology, burden of disease, and unmet needs in the treatment of ulcerative colitis in Asia. Expert Rev Gastroenterol Hepatol. 2021;15(3):275–89. 10.1080/17474124.2021.1840976. 33107344 10.1080/17474124.2021.1840976

[3] Aniwan S, Santiago P, Loftus EV Jr, Park SH. The epidemiology of inflammatory bowel disease in Asia and Asian immigrants to Western countries. United Eur Gastroenterol J. 2022;10(10):1063–76. 10.1002/ueg2.12350. 10.1002/ueg2.12350 PMC9752270 36479863

[4] Lewis JD, Parlett LE, Funk ML, Brensinger C, Pate V, Wu Q, Dawwas GK, Weiss A, Constant BD, McCauley M, Haynes K. Incidence, prevalence, and racial and ethnic distribution of inflammatory bowel disease in the United States. Gastroenterology. 2023;165(5):1197–205. 10.1053/j.gastro.2023.07.003. 37481117 10.1053/j.gastro.2023.07.003 PMC10592313

[5] Weber F, Eger KI, March C, Croner RS, Meyer F. Manifestation of acute appendicitis as known but paradox visceral side effect of ulcerative colitis anti-inflammatory therapy with januskinase-inhibitor Tofacitinib (Xeljanz™). Pathol Res Pract. 2023;248: 154333. 10.1016/j.prp.2023.154333. 37393666 10.1016/j.prp.2023.154333

[6] Gros B, Kaplan GG. Ulcerative colitis in adults: a review. JAMA. 2023;330(10):951–65. 10.1001/jama.2023.15389. 37698559 10.1001/jama.2023.15389

[7] Sandborn WJ, Danese S, Leszczyszyn J, Romatowski J, Altintas E, Peeva E, Hassan-Zahraee M, Vincent MS, Reddy PS, Banfield C, Salganik M. Oral ritlecitinib and brepocitinib for moderate-to-severe ulcerative colitis: results from a randomized, phase 2b study. Clin Gastroenterol Hepatol. 2023. 10.1016/j.cgh.2022.12.029. 36623678 10.1016/j.cgh.2022.12.029

[8] Lim J, Rezaie A. Irritable bowel syndrome-like symptoms in quiescent inflammatory bowel disease: a practical approach to diagnosis and treatment of organic causes. Dig Dis Sci. 2023;68(11):4081–97. 10.1007/s10620-023-08095-w. 37695549 10.1007/s10620-023-08095-w PMC10570178

[9] Singh S, Dulai PS. Ulcerative colitis: clinical manifestations and management. Yamada's Textbook of Gastroenterology. 2022:1248–93.10.1002/9781118512104.ch28

[10] Kotze PG, Heuthorst L, Lightner AL, Damião AO, Bemelman WA. New insights on the surgical management of ulcerative colitis in the 21st century. Lancet Gastroenterol Hepatol. 2022. 10.1016/S2468-1253(22)00001-2. 35364005 10.1016/S2468-1253(22)00001-2

[11] Raine T, Bonovas S, Burisch J, Kucharzik T, Adamina M, Annese V, Bachmann O, Bettenworth D, Chaparro M, Czuber-Dochan W, Eder P. ECCO guidelines on therapeutics in ulcerative colitis: medical treatment. J Crohns Colitis. 2022;16(1):2–17. 10.1093/ecco-jcc/jjab178. 34635919 10.1093/ecco-jcc/jjab178

[12] Guo XY, Liu XJ, Hao JY. Gut microbiota in ulcerative colitis: insights on pathogenesis and treatment. J Dig Dis. 2020;21(3):147–59. 10.1111/1751-2980.12849. 32040250 10.1111/1751-2980.12849

[13] Amiot A, Bouguen G, Bonnaud G, Bouhnik Y, Hagege H, Peyrin-Biroulet L, Abitbol V, Malamut G, Amiot A, Boruchowicz A, Siproudhis L. Clinical guidelines for the management of inflammatory bowel disease: update of a French national consensus. Dig Liver Dis. 2021;53(1):35–43. 10.1016/j.dld.2020.10.018. 33160886 10.1016/j.dld.2020.10.018

[14] Liu S, Eisenstein S. State-of-the-art surgery for ulcerative colitis. Langenbeck’s Arch Surg. 2021;406(6):1751–61. 10.1007/s00423-021-02295-6. 34453611 10.1007/s00423-021-02295-6 PMC8481179

[15] Lu L, Dong J, Liu Y, Qian Y, Zhang G, Zhou W, Zhao A, Ji G, Xu H. New insights into natural products that target the gut microbiota: effects on the prevention and treatment of colorectal cancer. Front Pharmacol. 2022;13: 964793. 10.3389/fphar.2022.964793. 36046819 10.3389/fphar.2022.964793 PMC9420899

[16] Salibay CC, Mahboob T, Verma AK, San Sebastian JS, Tabo HA, Raju CS, Nissapatorn V. Natural product–derived drugs for the treatment of inflammatory bowel diseases (IBD). Inflamm Nat Prod. 2021. 10.5217/ir.2014.12.2.103. 10.5217/ir.2014.12.2.103 PMC4204705 25349576

[17] Xue JC, Yuan S, Meng H, Hou XT, Li J, Zhang HM, Chen LL, Zhang CH, Zhang QG. The role and mechanism of flavonoid herbal natural products in ulcerative colitis. Biomed Pharmacother. 2023;158: 114086. 10.1016/j.biopha.2022.114086. 36502751 10.1016/j.biopha.2022.114086

[18] Duan L, Cheng S, Li L, Liu Y, Wang D, Liu G. Natural anti-inflammatory compounds as drug candidates for inflammatory bowel disease. Front Pharmacol. 2021;14(12): 684486. 10.3389/fphar.2021.684486. 10.3389/fphar.2021.684486 PMC8316996 34335253

[19] Akkol EK, Karpuz B, Sobarzo-Sánchez E, Khan H. A phytopharmacological overview of medicinal plants used for prophylactic and treatment of colitis. Food Chem Toxicol. 2020;144: 111628. 10.1016/j.fct.2020.111628. 32738379 10.1016/j.fct.2020.111628

[20] Dunleavy KA, Raffals LE, Camilleri M. Intestinal barrier dysfunction in inflammatory bowel disease: underpinning pathogenesis and therapeutics. Digest Dis Sci. 2023;68(12):4306–20. 10.1007/s10620-023-08122-w. 37773554 10.1007/s10620-023-08122-w PMC10798146

[21] Liu Z, Zhang Y, Jin T, Yi C, Ocansey DK, Mao F. The role of NOD2 in intestinal immune response and microbiota modulation: a therapeutic target in inflammatory bowel disease. Int Immunopharmacol. 2022;113: 109466. 10.1016/j.intimp.2022.109466. 36435061 10.1016/j.intimp.2022.109466

[22] Fu Q, Song T, Ma X, Cui J. Research progress on the relationship between intestinal microecology and intestinal bowel disease. Anim Models Exp Med. 2022;5(4):297–310. 10.1002/ame2.12262. 10.1002/ame2.12262 PMC9434592 35962562

[23] Chu J, Feng S, Guo C, Xue B, He K, Li L. Immunological mechanisms of inflammatory diseases caused by gut microbiota dysbiosis: s review. Biomed Pharmacother. 2023;164: 114985. 10.1016/j.biopha.2023.114985. 37311282 10.1016/j.biopha.2023.114985

[24] Mahapatro M, Erkert L, Becker C. Cytokine-mediated crosstalk between immune cells and epithelial cells in the gut. Cells. 2021;10(1):111. 10.3390/cells10010111. 33435303 10.3390/cells10010111 PMC7827439

[25] Nakase H, Sato N, Mizuno N, Ikawa Y. The influence of cytokines on the complex pathology of ulcerative colitis. Autoimmunity Rev. 2022;21(3): 103017. 10.1016/j.autrev.2021.103017. 34902606 10.1016/j.autrev.2021.103017

[26] Du L, Ha C. Epidemiology and pathogenesis of ulcerative colitis. Gastroenterol Clin. 2020;49(4):643–54. 10.1016/j.gtc.2020.07.005. 10.1016/j.gtc.2020.07.005 33121686

[27] Kaur A, Goggolidou P. Ulcerative colitis: understanding its cellular pathology could provide insights into novel therapies. J Inflamm. 2020;17:1–8. 10.1186/s12950-020-00246-4. 10.1186/s12950-020-00246-4 PMC7175540 32336953

[28] Kałużna A, Olczyk P, Komosińska-Vassev K. The role of innate and adaptive immune cells in the pathogenesis and development of the inflammatory response in ulcerative colitis. J Clin Med. 2022;11(2):400. 10.3390/jcm11020400. 35054093 10.3390/jcm11020400 PMC8780689

[29] Gomez-Bris R, Saez A, Herrero-Fernandez B, Rius C, Sanchez-Martinez H, Gonzalez-Granado JM. CD4 T-cell subsets and the pathophysiology of inflammatory bowel disease. Int J Mol Sci. 2023;24(3):2696. 10.3390/ijms24032696. 36769019 10.3390/ijms24032696 PMC9916759

[30] Tindemans I, Joosse ME, Samsom JN. Dissecting the heterogeneity in T-cell mediated inflammation in IBD. Cells. 2020;9(1):110. 10.3390/cells9010110. 31906479 10.3390/cells9010110 PMC7016883

[31] Lu Q, Yang MF, Liang YJ, Xu J, Xu HM, Nie YQ, Wang LS, Yao J, Li DF. Immunology of inflammatory bowel disease: molecular mechanisms and therapeutics. J Inflamm Res. 2022;15:1825–44. 10.2147/JIR.S353038. 35310454 10.2147/JIR.S353038 PMC8928114

[32] Moreira Lopes TC, Mosser DM, Gonçalves R. Macrophage polarization in intestinal inflammation and gut homeostasis. Inflamm Res. 2020;69:1163–72. 10.1007/s00011-020-01398-y. 32886145 10.1007/s00011-020-01398-y

[33] Yip JL, Balasuriya GK, Spencer SJ, Hill-Yardin EL. The role of intestinal macrophages in gastrointestinal homeostasis: heterogeneity and implications in disease. Cell Mol Gastroenterol Hepatol. 2021;12(5):1701–18. 10.1016/j.jcmgh.2021.08.021. 34506953 10.1016/j.jcmgh.2021.08.021 PMC8551786

[34] Xiang C, Liu M, Lu Q, Fan C, Lu H, Feng C, Yang X, Li H, Tang W. Blockade of TLRs-triggered macrophage activation by caffeic acid exerted protective effects on experimental ulcerative colitis. Cell Immunol. 2021;365: 104364. 10.1016/j.cellimm.2021.104364. 33932876 10.1016/j.cellimm.2021.104364

[35] Dharmasiri S, Garrido-Martin EM, Harris RJ, Bateman AC, Collins JE, Cummings JF, Sanchez-Elsner T. Human intestinal macrophages are involved in the pathology of both ulcerative colitis and Crohn disease. Inflamm Bowel Dis. 2021;27(10):1641–52. 10.1093/ibd/izab029. 33570153 10.1093/ibd/izab029 PMC8522792

[36] Gohil S, Majd Z, Sheneman JC, Abughosh SM. Interventions to improve medication adherence in inflammatory bowel disease: a systematic review. Patient Educ Counsel. 2022;105(7):1731–42. 10.1016/j.pec.2021.10.017. 10.1016/j.pec.2021.10.017 34736829

[37] Gajendran M, Loganathan P, Jimenez G, Catinella AP, Ng N, Umapathy C, Ziade N, Hashash JG. A comprehensive review and update on ulcerative colitis. Dis Mon. 2019;65(12): 100851. 10.1016/j.disamonth.2019.02.004. 30837080 10.1016/j.disamonth.2019.02.004

[38] Smith RL, Taylor KM, Friedman AB, Gibson RN, Gibson PR. Systematic review: clinical utility of gastrointestinal ultrasound in the diagnosis, assessment and management of patients with ulcerative colitis. J Crohns Colitis. 2020;14(4):465–79. 10.1093/ecco-jcc/jjz163. 31562739 10.1093/ecco-jcc/jjz163

[39] Kucharzik T, Koletzko S, Kannengiesser K, Dignass A. Ulcerative colitis—diagnostic and therapeutic algorithms. Dtsch Arztebl Int. 2020;117(33–34):564. 10.3238/arztebl.2020.0564. 33148393 10.3238/arztebl.2020.0564 PMC8171548

[40] Axelrad JE, Olén O, Askling J, Lebwohl B, Khalili H, Sachs MC, Ludvigsson JF. Gastrointestinal infection increases odds of inflammatory bowel disease in a nationwide case–control study. Clin Gastroenterol Hepatol. 2019;17(7):1311–22. 10.1016/j.cgh.2018.09.034. 30389589 10.1016/j.cgh.2018.09.034

[41] Frickenstein AN, Jones MA, Behkam B, McNally LR. Imaging inflammation and infection in the gastrointestinal tract. Int J Mol Sci. 2019;21(1):243. 10.3390/ijms21010243. 31905812 10.3390/ijms21010243 PMC6981656

[42] Takenaka K, Fujii T, Kawamoto A, Suzuki K, Shimizu H, Maeyashiki C, Yamaji O, Motobayashi M, Igarashi A, Hanazawa R, Hibiya S. Deep neural network for video colonoscopy of ulcerative colitis: a cross-sectional study. Lancet Gastroenterol Hepatol. 2022;7(3):230–7. 10.1016/S2468-1253(21)00372-1. 34856196 10.1016/S2468-1253(21)00372-1

[43] Mokter MF, Oh J, Tavanapong W, Wong J, de Groen PC. Classification of ulcerative colitis severity in colonoscopy videos using vascular pattern detection. InMachine Learning in Medical Imaging: 11th International Workshop, MLMI 2020, Held in Conjunction with MICCAI 2020, Lima, Peru, October 4, 2020, Proceedings 11 2020 (pp. 552–562). Springer International Publishing. 10.1007/978-3-030-59861-7_56

[44] Linggi B, Jairath V, Zou G, Shackelton LM, McGovern DP, Salas A, Verstockt B, Silverberg MS, Nayeri S, Feagan BG, van de Casteele N. Meta-analysis of gene expression disease signatures in colonic biopsy tissue from patients with ulcerative colitis. Sci Rep. 2021;11(1):18243. 10.1038/s41598-021-97366-5. 34521888 10.1038/s41598-021-97366-5 PMC8440637

[45] Battat R, Vande Casteele N, Pai RK, Wang Z, Zou G, McDonald JW, Duijvestein M, Jeyarajah J, Parker CE, Van Viegen T, Nelson SA. Evaluating the optimum number of biopsies to assess histological inflammation in ulcerative colitis: a retrospective cohort study. Aliment Pharmacol Ther. 2020;52(10):1574–82. 10.1111/apt.16083. 32981088 10.1111/apt.16083 PMC8007067

[46] Park SB, Kim SJ, Lee J, Lee YJ, Baek DH, Seo GS, Kim ES, Kim SW, Kim SY. Efficacy of sigmoidoscopy for evaluating disease activity in patients with ulcerative colitis. BMC Gastroenterol. 2022;22(1):1–7. 10.1186/s12876-022-02178-0. 35220941 10.1186/s12876-022-02178-0 PMC8882296

[47] Chen H, Wu L, Wang M, Shao B, Ye L, Zhang Y, Cao Q. Use of the ulcerative colitis endoscopic index of severity and Mayo endoscopic score for predicting the therapeutic effect of mesalazine in patients with ulcerative colitis. Laparosc Endosc Robot Surg. 2021;4(2):33–9. 10.1016/j.lers.2021.04.003. 10.1016/j.lers.2021.04.003

[48] Pagnini C, Di Paolo MC, Mariani BM, Urgesi R, Pallotta L, Vitale MA, Villotti G, d’Alba L, De Cesare MA, Di Giulio E, Graziani MG. Mayo endoscopic score and ulcerative colitis endoscopic index are equally effective for endoscopic activity evaluation in ulcerative colitis patients in a real-life setting. Gastroenterol Insights. 2021;12(2):217–24. 10.3390/gastroent12020019. 10.3390/gastroent12020019

[49] Xiao BH, Ma XD, Lv JJ, Yang T, Liu XJ, An LY, Qi YX, Lu ML, Duan YQ, Sun DL. Systematic evaluation of the diagnostic approach of inflammatory bowel disease guidelines. Int J Clin Pract. 2021;75(10):14365. 10.1111/ijcp.14365. 10.1111/ijcp.14365 34008296

[50] Shaban N, Hoad CL, Naim I, Alshammari M, Radford SJ, Clarke C, Marciani L, Moran G. Imaging in inflammatory bowel disease: current and future perspectives. Frontline Gastroenterol. 2022. 10.1136/flgastro-2022-102117. 35812031 10.1136/flgastro-2022-102117 PMC9234729

[51] Le Berre C, Ananthakrishnan AN, Danese S, Singh S, Peyrin-Biroulet L. Ulcerative colitis and Crohn’s disease have similar burden and goals for treatment. Clin Gastroenterol Hepatol. 2020;18(1):14–23. 10.1016/j.cgh.2019.07.005. 31301452 10.1016/j.cgh.2019.07.005

[52] D’Amico F, Fasulo E, Jairath V, Paridaens K, Peyrin-Biroulet L, Danese S. Management and treatment optimization of patients with mild to moderate ulcerative colitis. Expert Rev Clin Immunol. 2023;13:1–4. 10.1080/1744666X.2023.2292768. 10.1080/1744666X.2023.2292768 38059454

[53] Cai Z, Wang S, Li J. Treatment of inflammatory bowel disease: a comprehensive review. Front Med. 2021;8: 765474. 10.3389/fmed.2021.765474. 10.3389/fmed.2021.765474 PMC8720971 34988090

[54] Chen M, Lan H, Jin K, Chen Y. Responsive nanosystems for targeted therapy of ulcerative colitis: current practices and future perspectives. Drug Deliv. 2023;30(1):2219427. 10.1080/10717544.2023.2219427. 37288799 10.1080/10717544.2023.2219427 PMC10405869

[55] Ferretti F, Cannatelli R, Monico MC, Maconi G, Ardizzone S. An update on current pharmacotherapeutic options for the treatment of ulcerative colitis. J Clin Med. 2022;11(9):2302. 10.3390/jcm11092302. 35566428 10.3390/jcm11092302 PMC9104748

[56] Alsoud D, Verstockt B, Fiocchi C, Vermeire S. Breaking the therapeutic ceiling in drug development in ulcerative colitis. Lancet Gastroenterol Hepatol. 2021;6(7):589–95. 10.1016/S2468-1253(21)00065-0. 34019798 10.1016/S2468-1253(21)00065-0

[57] Gupta M, Mishra V, Gulati M, Kapoor B, Kaur A, Gupta R, Tambuwala MM. Natural compounds as safe therapeutic options for ulcerative colitis. Inflammopharmacology. 2022;30(2):397–434. 10.1007/s10787-022-00931-1. 35212849 10.1007/s10787-022-00931-1 PMC8948151

[58] Moudgil KD, Venkatesha SH. The anti-inflammatory and immunomodulatory activities of natural products to control autoimmune inflammation. Int J Mol Sci. 2022;24(1):95. 10.3390/ijms24010095. 36613560 10.3390/ijms24010095 PMC9820125

[59] El Menyiy N, El Allam A, Aboulaghras S, Jaouadi I, Bakrim S, El Omari N, Shariati MA, Miftakhutdinov A, Wilairatana P, Mubarak MS, Bouyahya A. Inflammatory auto-immune diseases of the intestine and their management by natural bioactive compounds. Biomed Pharmacother. 2022;1(151): 113158. 10.1016/j.biopha.2022.113158. 10.1016/j.biopha.2022.113158 35644116

[60] Davila MM, Papada E. The role of plant-derived natural products in the management of inflammatory bowel disease—what is the clinical evidence so far? Life. 2023;13(8):1703. 10.1586/17474124.2016.1145546. 37629560 10.1586/17474124.2016.1145546 PMC10455079

[61] Guo N, Lv LL. Mechanistic insights into the role of probiotics in modulating immune cells in ulcerative colitis. Immunity, Inflamm Dis. 2023;11(10): e1045. 10.1002/iid3.1045. 10.1002/iid3.1045 PMC10571014 37904683

[62] Chang Y, Zhai L, Peng J, Wu H, Bian Z, Xiao H. Phytochemicals as regulators of Th17/Treg balance in inflammatory bowel diseases. Biomed Pharmacother. 2021;1(141): 111931. 10.1016/j.biopha.2021.111931. 10.1016/j.biopha.2021.111931 34328111

[63] Chen L, Ruan G, Cheng Y, Yi A, Chen D, Wei Y. The role of Th17 cells in inflammatory bowel disease and the research progress. Front Immunol. 2023;9(13):1055914. 10.3389/fimmu.2022.1055914. 10.3389/fimmu.2022.1055914 PMC9870314 36700221

[64] Zhao J, Lu Q, Liu Y, Shi Z, Hu L, Zeng Z, Tu Y, Xiao Z, Xu Q. Th17 cells in inflammatory bowel disease: cytokines, plasticity, and therapies. J Immunol Res. 2021;22:2021. 10.1155/2021/8816041. 10.1155/2021/8816041 PMC7846404 33553436

[65] Caioni G, Viscido A, Angelo M, Panella G, Castelli V, Merola C, Frieri G, Latella G, Cimini A, Benedetti E. Inflammatory bowel disease: new insights into the interplay between environmental factors and PPARγ. Int J Mol Sci. 2021;22(3):985. 10.3390/ijms22030985. 33498177 10.3390/ijms22030985 PMC7863964

[66] Villarroel-Vicente C, Gutierrez-Palomo S, Ferri J, Cortes D, Cabedo N. Natural products and analogs as preventive agents for metabolic syndrome via peroxisome proliferator-activated receptors: an overview. Eur J Med Chem. 2021;5(221): 113535. 10.1016/j.ejmech.2021.113535. 10.1016/j.ejmech.2021.113535 33992930

[67] Venkataraman B, Ojha S, Belur PD, Bhongade B, Raj V, Collin PD, Adrian TE, Subramanya SB. Phytochemical drug candidates for the modulation of peroxisome proliferator-activated receptor γ in inflammatory bowel diseases. Phytother Res. 2020;34(7):1530–49. 10.1002/ptr.6625. 32009281 10.1002/ptr.6625

[68] Laurindo LF, de Maio MC, Minniti G, de Góes CN, Barbalho SM, Quesada K, Guiguer EL, Sloan KP, Detregiachi CR, Araújo AC, de Alvares GR. Effects of medicinal plants and phytochemicals in Nrf2 pathways during inflammatory bowel diseases and related colorectal cancer: a comprehensive review. Metabolites. 2023;13(2):243. 10.3390/metabo13020243. 36837862 10.3390/metabo13020243 PMC9966918

[69] Liu H, Johnston LJ, Wang F, Ma X. Triggers for the nrf2/are signaling pathway and its nutritional regulation: Potential therapeutic applications of ulcerative colitis. Int J Mol Sci. 2021;22(21):11411. 10.1177/17562848221138160. 34768841 10.1177/17562848221138160 PMC8583850

[70] Laurindo LF, Santos AR, Carvalho AC, Bechara MD, Guiguer EL, Goulart RD, Vargas Sinatora R, Araújo AC, Barbalho SM. Phytochemicals and regulation of NF-kB in inflammatory bowel diseases: an overview of in vitro and in vivo effects. Metabolites. 2023;13(1):96. 10.3390/metabo13010096. 36677021 10.3390/metabo13010096 PMC9862976

[71] Yu C, Wang D, Yang Z, Wang T. Pharmacological effects of polyphenol phytochemicals on the intestinal inflammation via targeting TLR4/NF-κB signaling pathway. Int J Mol Sci. 2022;23(13):6939. 10.3390/ijms23136939. 35805952 10.3390/ijms23136939 PMC9266441

[72] Wang K, Mao T, Lu X, Wang M, Yun Y, Jia Z, Shi L, Jiang H, Li J, Shi R. A potential therapeutic approach for ulcerative colitis: targeted regulation of macrophage polarization through phytochemicals. Front Immunol. 2023;1(14):1155077. 10.3389/fimmu.2023.1155077. 10.3389/fimmu.2023.1155077 PMC10183582 37197668

[73] Du Y, Rong L, Cong Y, Shen L, Zhang N, Wang B. Macrophage polarization: an effective approach to targeted therapy of inflammatory bowel disease. Expert Opin Ther Targets. 2021;25(3):191–209. 10.1080/14728222.2021.1901079. 33682588 10.1080/14728222.2021.1901079

[74] do Nascimento RD, da FonsecaMachado AP, Galvez J, Cazarin CB, Junior MR. Ulcerative colitis: gut microbiota, immunopathogenesis and application of natural products in animal models. Life Sci. 2020;258: 118129. 10.1016/j.lfs.2020.118129. 32717271 10.1016/j.lfs.2020.118129

[75] Abe H, Ishioka M, Fujita Y, Umeno A, Yasunaga M, Sato A, Ohnishi S, Suzuki S, Ishida N, Shichiri M, Yoshida Y. Yuzu (*Citrus junos* Tanaka) peel attenuates dextran sulfate sodium-induced murine experimental colitis. J Oleo Sci. 2018;67(3):335–44. 10.5650/jossess17184. 29459515 10.5650/jossess17184

[76] Shin J, Song HY, Lee M. Sudachinoid-and ichangensin-type limonoids from *Citrus junos* downregulate pro-inflammatory cytokines. Int J Mol Sci. 2020;21(18):6963. 10.3390/ijms21186963. 32971925 10.3390/ijms21186963 PMC7555237

[77] Kim SH, Shin EJ, Hur HJ, Park JH, Sung MJ, Kwon DY, Hwang JT. *Citrus junos* Tanaka peel extract attenuates experimental colitis and inhibits tumour growth in a mouse xenograft model. J Funct Foods. 2014;8:301–8. 10.1016/j.jff.2014.03.024. 10.1016/j.jff.2014.03.024

[78] Kim Y, Wu AG, Jaja-Chimedza A, Graf BL, Waterman C, Verzi MP, Raskin I. Isothiocyanate-enriched moringa seed extract alleviates ulcerative colitis symptoms in mice. PLoS ONE. 2017;12(9): e0184709. 10.1371/journal.pone.0184709. 28922365 10.1371/journal.pone.0184709 PMC5602518

[79] Mohamed Husien H, Peng W, Su H, Zhou R, Tao Y, Huang J, Liu M, Bo R, Li J. *Moringa oleifera* leaf polysaccharide alleviates experimental colitis by inhibiting inflammation and maintaining intestinal barrier. Front Nutr. 2022;10(9):1055791. 10.3389/fnut.2022.1055791. 10.3389/fnut.2022.1055791 PMC9686441 36438754

[80] Hong ZS, Xie J, Wang XF, Dai JJ, Mao JY, Bai YY, Sheng J, Tian Y. *Moringa oleifera* Lam. peptide remodels intestinal mucosal barrier by inhibiting JAK-STAT activation and modulating gut microbiota in colitis. Front Immunol. 2022;13: 924178. 10.3389/fimmu.2022.924178. 35911761 10.3389/fimmu.2022.924178 PMC9336532

[81] Zhang Y, Peng L, Li W, Dai T, Nie L, Xie J, Ai Y, Li L, Tian Y, Sheng J. Polyphenol extract of *Moringa oleifera* leaves alleviates colonic inflammation in dextran sulfate sodium-treated mice. Evid Based Complement Altern Med. 2020. 10.1155/2020/6295402. 10.1155/2020/6295402 PMC7710425 33299453

[82] Shao S, Wang D, Zheng W, Li X, Zhang H, Zhao D, Wang M. A unique polysaccharide from *Hericium erinaceus* mycelium ameliorates acetic acid-induced ulcerative colitis rats by modulating the composition of the gut microbiota, short chain fatty acids levels and GPR41/43 respectors. Int Immunopharmacol. 2019;1(71):411–22. 10.1016/j.intimp.2019.02.038. 10.1016/j.intimp.2019.02.038 31059977

[83] Wang D, Zhang Y, Yang S, Zhao D, Wang M. A polysaccharide from cultured mycelium of *Hericium erinaceus* relieves ulcerative colitis by counteracting oxidative stress and improving mitochondrial function. Int J Biol Macromol. 2019;125:572–9. 10.1016/j.ijbiomac.2018.12.092. 30543884 10.1016/j.ijbiomac.2018.12.092

[84] Ren Y, Sun Q, Gao R, Sheng Y, Guan T, Li W, Zhou L, Liu C, Li H, Lu Z, Yu L. Low weight polysaccharide of *Hericium erinaceus* ameliorates colitis via inhibiting the NLRP3 inflammasome activation in association with gut microbiota modulation. Nutrients. 2023;15(3):739. 10.3390/nu15030739. 36771444 10.3390/nu15030739 PMC9920828

[85] Durmus A, Durmus I, Bender O, Karatepe O. The effect of *Hericium erinaceum* on the prevention of chemically induced experimental colitis in rats. Korean J Intern Med. 2021;36(Suppl 1):S44. 10.3904/kjim.2019.050. 32550720 10.3904/kjim.2019.050 PMC8009150

[86] Gravina AG, Pellegrino R, Palladino G, Coppola A, Brandimarte G, Tuccillo C, Ciardiello F, Romano M, Federico A. Hericium erinaceus, in combination with natural flavonoid/alkaloid and B3/B8 vitamins, can improve inflammatory burden in Inflammatory bowel diseases tissue: an ex vivo study. Front Immunol. 2023. 10.3389/fimmu.2023.1215329. 37465689 10.3389/fimmu.2023.1215329 PMC10350490

[87] Shah TA, Parikh M, Patel KV, Patel KG, Joshi CG, Gandhi TR. Evaluation of the effect of *Punica granatum* juice and punicalagin on NFκB modulation in inflammatory bowel disease. Mol Cell Biochem. 2016;419:65–74. 10.1007/s11010-016-2750-x. 27352379 10.1007/s11010-016-2750-x

[88] Kamali M, Tavakoli H, Khodadoost M, Daghaghzadeh H, Kamalinejad M, Gachkar L, Mansourian M, Adibi P. Efficacy of the *Punica granatum* peels aqueous extract for symptom management in ulcerative colitis patients. A randomized, placebo-controlled, clinical trial. Complement Ther Clin Pract. 2015;21(3):141–6. 10.1016/j.ctcp.2015.03.001. 26256131 10.1016/j.ctcp.2015.03.001

[89] Kalyankumarraju M, Puppala ER, Ahmed S, Kumar GJ, Tene K, Syamprasad NP, Sahu BD, Barua CC, Naidu VG. *Zanthoxylum alatum* Roxb. seed extract ameliorates stress aggravated DSS-induced ulcerative colitis in mice: plausible role on NF-κB signaling axis. J Ethnopharmacol. 2021;279: 114385. 10.1016/j.jep.2021.114385. 34217795 10.1016/j.jep.2021.114385

[90] Zaware B, Gilhotra R, Chaudhari SR. Potential of *Mimosa pudica* leaf in the treatment of ulcerative colitis in rat. Bangladesh J Pharmacol. 2018;13(3):241–7. 10.3329/bjp.v13i3.35648. 10.3329/bjp.v13i3.35648

[91] Recinella L, Gorica E, Chiavaroli A, Fraschetti C, Filippi A, Cesa S, Cairone F, Martelli A, Calderone V, Veschi S, Lanuti P. Anti-inflammatory and antioxidant effects induced by *Allium sativum* L. extracts on an ex vivo experimental model of ulcerative colitis. Foods. 2022;11(22):3559. 10.3390/foods11223559. 36429152 10.3390/foods11223559 PMC9689397

[92] Owusu G, Obiri DD, Ainooson GK, Osafo N, Antwi AO, Duduyemi BM, Ansah C. Acetic acid-induced ulcerative colitis in Sprague Dawley rats is suppressed by hydroethanolic extract of *Cordia vignei* leaves through reduced serum levels of TNF-α and IL-6. Int J Chronic Dis. 2020;6:2020. 10.1155/2020/8785497. 10.1155/2020/8785497 PMC7026722 32090060

[93] Adjouzem CF, Gilbert A, Mbiantcha M, Yousseu Nana W, Matah Marthe Mba V, Djuichou Nguemnang SF, Tsafack EG, Atsamo AD. Effects of aqueous and methanolic extracts of stem bark of Ansonia bonnie De wild (A polynucleate) on dextran sodium sulfate-induced ulcerative colitis in Wistar rats. Evid Based Complement Altern Med. 2020. 10.1155/2020/4918453. 10.1155/2020/4918453 PMC7277065 32565862

[94] Qin W, Luo H, Yang L, Hu D, Jiang SP, Peng DY, Hu JM, Liu SJ. *Rubia cordifolia* L. ameliorates DSS-induced ulcerative colitis in mice through dual inhibition of NLRP3 inflammasome and IL-6/JAK2/STAT3 pathways. Heliyon. 2022. 10.1016/j.heliyon.2022.e10314. 36082330 10.1016/j.heliyon.2022.e10314 PMC9445285

[95] Gupta RA, Motiwala MN, Mahajan UN, Sabre SG. Protective effect of *Sesbania grandiflora* on acetic acid induced ulcerative colitis in mice by inhibition of TNF-α and IL-6. J Ethnopharmacol. 2018;12(219):222–32. 10.1016/j.jep.2018.02.043. 10.1016/j.jep.2018.02.043 29530609

[96] Meurer MC, Mees M, Mariano LN, Boeing T, Somensi LB, Mariott M, Dos Santos AC, Longo B, França TC, Klein-Júnior LC, de Souza P. Hydroalcoholic extract of *Tagetes erecta* L. flowers, rich in the carotenoid lutein, attenuates inflammatory cytokine secretion and improves the oxidative stress in an animal model of ulcerative colitis. Nutr Res. 2019;66:95–106. 10.1016/j.nutres.2019.03.005. 30979660 10.1016/j.nutres.2019.03.005

[97] Almeer RS, Mahmoud SM, Amin HK, Moneim AE. *Ziziphus spina-christi* fruit extract suppresses oxidative stress and p38 MAPK expression in ulcerative colitis in rats via induction of Nrf2 and HO-1 expression. Food Chem Toxicol. 2018;1(115):49–62. 10.1016/j.fct.2018.03.002. 10.1016/j.fct.2018.03.002 29518435

[98] Suluvoy JK, Sakthivel KM, Guruvayoorappan C, Grace VB. Protective effect of *Averrhoa bilimbi* L. fruit extract on ulcerative colitis in wistar rats via regulation of inflammatory mediators and cytokines. Biomed Pharmacother. 2017;91:1113–21. 10.1016/j.biopha.2017.05.057. 28531922 10.1016/j.biopha.2017.05.057

[99] Chen G, Yang Y, Liu M, Teng Z, Ye J, Xu Y, Cai X, Cheng X, Yang J, Hu C, Wang M. Banxia xiexin decoction protects against dextran sulfate sodium-induced chronic ulcerative colitis in mice. J Ethnopharmacol. 2015;26(166):149–56. 10.1016/j.jep.2015.03.027. 10.1016/j.jep.2015.03.027 25794808

[100] Liu Y, Wang C, Wu J, Tan L, Gao P, Wu S, Tang D, Wang Q, Wang C, Li P, Liu J. Study on the comprehensive phytochemicals and the anti-ulcerative colitis effect of *Saussurea pulchella*. Molecules. 2023;28(4):1526. 10.3390/molecules28041526. 36838515 10.3390/molecules28041526 PMC9964537

[101] Rehman IU, Saleem M, Raza SA, Bashir S, Muhammad T, Asghar S, Qamar MU, Shah TA, Bin Jardan YA, Mekonnen AB, Bourhia M. Anti-ulcerative colitis effects of chemically characterized extracts from *Calliandra haematocephala* in acetic acid-induced ulcerative colitis. Front Chem. 2024;27(12):1291230. 10.3389/fchem.2024.1291230. 10.3389/fchem.2024.1291230 PMC10927971 38476652

[102] Devi K, Bali A, Bhatia P, Singh N, Jaggi AS. Exploring the ameliorative potential of *Bacopa monnieri* in acetic acid induced ulcerative colitis in mice. Nat Prod Res. 2024;38(12):2105–10. 10.3389/fchem.2024.1291230. 37427984 10.3389/fchem.2024.1291230

[103] Jia W, Yu S, Liu X, Le Q, He X, Yu L, He J, Yang L, Gao H. Ethanol extract of limonium bicolor improves dextran sulfate sodium-induced ulcerative colitis by alleviating inflammation and restoring gut microbiota dysbiosis in mice. Mar Drugs. 2024;22(4):175. 10.3390/md22040175. 38667792 10.3390/md22040175 PMC11050939

[104] Abdellatif NA, Eltamany EE, El-Shenawy NS, Nafie MS, Hassan YM, Al-Eisa RA, Badr JM, Abdelhameed RF. *Cassia fistula* leaves extract profiling and its emphasis on induced ulcerative colitis in male rats through inhibition of caspase 3 and cyclooxygenase-2. Arab J Chem. 2024;16: 105672. 10.1016/j.arabjc.2024.105672. 10.1016/j.arabjc.2024.105672

[105] Zhao Q, Zhu L, Wang S, Gao Y, Jin F. Molecular mechanism of the anti-inflammatory effects of plant essential oils: a systematic review. J Ethnopharmacol. 2023;30(301): 115829. 10.1016/j.jep.2022.115829. 10.1016/j.jep.2022.115829 36252876

[106] Li J, Zhang X, Luan F, Duan J, Zou J, Sun J, Shi Y, Guo D, Wang C, Wang X. Therapeutic potential of essential oils against ulcerative colitis: a review. J Inflamm Res. 2024;17:3527. 10.2147/JIR.S461466. 38836243 10.2147/JIR.S461466 PMC11149639

[107] de Santana Souza MT, Teixeira DF, de Oliveira JP, Oliveira AS, Quintans-Junior LJ, Correa CB, Camargo EA. Protective effect of carvacrol on acetic acid-induced colitis. Biomed Pharmacother. 2017;1(96):313–9. 10.1016/j.biopha.2017.10.017. 10.1016/j.biopha.2017.10.017 29017143

[108] Liu M, Mao G, Zhou X, Wan X, Zhang F, Dai L, Chen Y, Dai N, Zhang Y, Du Q. Carvacrol ameliorates DSS-induced intestinal inflammation in rats by suppressing the TLR4/NF-κB pathway. Nat Prod Commun. 2022. 10.1177/1934578X221130147.35136386 10.1177/1934578X221130147

[109] d’Alessio PA, Ostan R, Bisson JF, Schulzke JD, Ursini MV, Béné MC. Oral administration of d-limonene controls inflammation in rat colitis and displays anti-inflammatory properties as diet supplementation in humans. Life Sci. 2013;92(24–26):1151–6. 10.1016/j.lfs.2013.04.013. 23665426 10.1016/j.lfs.2013.04.013

[110] Yu L, Yan J, Sun Z. D-limonene exhibits anti-inflammatory and antioxidant properties in an ulcerative colitis rat model via regulation of iNOS, COX-2, PGE2 and ERK signaling pathways. Mol Med Rep. 2017;15(4):2339–46. 10.3892/mmr.2017.6241. 28260017 10.3892/mmr.2017.6241

[111] Estrella GR, Eva GT, Alberto HL, Guadalupe VD, Azucena CV, Sandra OS, Noé AV, Javier LM. Limonene from *Agastache mexicana* essential oil produces antinociceptive effects, gastrointestinal protection and improves experimental ulcerative colitis. J Ethnopharmacol. 2021;15(280): 114462. 10.1016/j.jep.2021.114462. 10.1016/j.jep.2021.114462 34324951

[112] Liu DM, Zhou CY, Meng XL, Wang P, Li W. Thymol exerts anti-inflammatory effect in dextran sulfate sodium-induced experimental murine colitis. Trop J Pharm Res. 2018;17(9):1803–10. 10.4314/tjpr.v17i9.18. 10.4314/tjpr.v17i9.18

[113] Chamanara M, Abdollahi A, Rezayat SM, Ghazi-Khansari M, Dehpour A, Nassireslami E, Rashidian A. Thymol reduces acetic acid-induced inflammatory response through inhibition of NF-kB signaling pathway in rat colon tissue. Inflammopharmacology. 2019;27:1275–83. 10.1007/s10787-019-00583-8. 30903350 10.1007/s10787-019-00583-8

[114] Tahmasebi P, Froushani SM, Ahangaran NA. Thymol has beneficial effects on the experimental model of ulcerative colitis. Avicenna J Phytomed. 2019;9(6):538. 10.22038/AJP.2019.13383. 31763213 10.22038/AJP.2019.13383 PMC6823534

[115] Ghasemi-Pirbaluti M, Motaghi E, Bozorgi H. The effect of menthol on acute experimental colitis in rats. Eur J Pharmacol. 2017;15(805):101–7. 10.1016/j.ejphar.2017.03.003. 10.1016/j.ejphar.2017.03.003 28322843

[116] Bastaki SM, Adeghate E, Amir N, Ojha S, Oz M. Menthol inhibits oxidative stress and inflammation in acetic acid-induced colitis in rat colonic mucosa. Am J Transl Res. 2018;10(12):4210. 30662664 PMC6325525

[117] Luo L, Yan J, Chen B, Luo Y, Liu L, Sun Z, Lu Y. The effect of menthol supplement diet on colitis-induced colon tumorigenesis and intestinal microbiota. Am J Transl Res. 2021;13(1):38. 33527007 PMC7847519

[118] Ma S, Yang B, Du Y, Lv Y, Liu J, Shi Y, Huang T, Xu H, Deng L, Chen X. 1, 8-cineole ameliorates colon injury by downregulating macrophage M1 polarization via inhibiting the HSP90-NLRP3-SGT1 complex. J Pharm Anal. 2023;13(9):984–98. 10.1016/j.jpha.2023.07.001. 37842654 10.1016/j.jpha.2023.07.001 PMC10568110

[119] Venkataraman B, Almarzooqi S, Raj V, Bhongade BA, Patil RB, Subramanian VS, Attoub S, Rizvi TA, Adrian TE, Subramanya SB. Molecular docking identifies 1, 8-Cineole (Eucalyptol) as a novel PPARγ agonist that alleviates colon inflammation. Int J Mol Sci. 2023;24(7):6160. 10.3390/ijms24076160. 37047133 10.3390/ijms24076160 PMC10094723

[120] Subramanya SB, Venkataraman B, Almarzooqi S, Raj V, Subramanian VS, Bhongade BA. 1, 8-Cineole, a bioactive monoterpenoid, mitigates colon inflammation by stimulating colon PPAR-γ transcription factor. FASEB J. 2022. 10.1096/fasebj.2022.36.S1.R3110. 10.1096/fasebj.2022.36.S1.R3110

[121] Hossen I, Hua W, Ting L, Mehmood A, Jingyi S, Duoxia X, Yanping C, Hongqing W, Zhipeng G, Kaiqi Z, Fang Y. Phytochemicals and inflammatory bowel disease: a review. Crit Rev Food Sci Nutr. 2022;60(8):1321–45. 10.1080/10408398.2019.1570913. 10.1080/10408398.2019.1570913 30729797

[122] Moon SY, Kim KD, Yoo J, Lee JH, Hwangbo C. Phytochemicals targeting JAK–STAT pathways in inflammatory bowel disease: insights from animal models. Molecules. 2021;26(9):2824. 10.3390/molecules26092824. 34068714 10.3390/molecules26092824 PMC8126249

[123] Zhang J, Sun S, Chen H, Feng Y, Li Y, Dong Z. Advances in natural compound-based nanomedicine and the interaction with gut microbiota in ulcerative colitis therapy. Front Pharmacol. 2023. 10.3389/fphar.2023.1197144. 37521480 10.3389/fphar.2023.1197144 PMC10372797

[124] Zhang H, Lang W, Li S, Xu C, Wang X, Li Y, Zhang Z, Wu T, Feng M. Corynoline ameliorates dextran sulfate sodium-induced colitis in mice by modulating Nrf2/NF-κB pathway. Immunopharmacol Immunotoxicol. 2023;45(1):26–34. 10.1080/08923973.2022.2112218. 35980837 10.1080/08923973.2022.2112218

[125] Zhao M, Li P, Qiao D, Hua S, Yue Q, Dai Y, Huang Y, Jiang J, Yin H, Li M, Ding Y. N6-methyladenosine modification of TSC1 mRNA contributes to macrophage polarization regulated by Coptisine in DSS-induced ulcerative colitis. Phytomedicine. 2024;1(122): 155153. 10.1016/j.phymed.2023.155153. 10.1016/j.phymed.2023.155153 38014839

[126] Zhang MN, Xie R, Wang HG, Wen X, Wang JY, He L, Zhang MH, Yang XZ. Cepharanthine alleviates DSS-induced ulcerative colitis via regulating aconitate decarboxylase 1 expression and macrophage infiltration. Molecules. 2023;28(3):1060. 10.3390/molecules28031060. 36770726 10.3390/molecules28031060 PMC9920045

[127] Yue M, Huang J, Ma X, Huang P, Liu Y, Zeng J. Protopine alleviates dextran sodium sulfate-induced ulcerative colitis by improving intestinal barrier function and regulating intestinal microbiota. Molecules. 2023;28(13):5277. 10.3390/molecules28135277. 37446938 10.3390/molecules28135277 PMC10343248

[128] Liu C, Wang R, Jiao X, Zhang J, Zhang C, Wang Z. Oxysophocarpine suppresses TRAF6 level to ameliorate oxidative stress and inflammatory factors secretion in mice with dextran sulphate sodium (DSS) induced-ulcerative colitis. Microb Pathog. 2023;1(182): 106244. 10.1016/j.micpath.2023.106244. 10.1016/j.micpath.2023.106244 37423495

[129] Ekhtiar M, Ghasemi-Dehnoo M, Mirzaei Y, Azadegan-Dehkordi F, Amini-Khoei H, Lorigooini Z, Samiei-Sefat A, Bagheri N. The coumaric acid and syringic acid ameliorate acetic acid-induced ulcerative colitis in rats via modulator of Nrf2/HO-1 and pro-inflammatory cytokines. Int Immunopharmacol. 2023;1(120): 110309. 10.1016/j.intimp.2023.110309. 10.1016/j.intimp.2023.110309 37182450

[130] Prakash T, Janadri S. Anti-inflammatory effect of wedelolactone on DSS induced colitis in rats: IL-6/STAT3 signaling pathway. J Ayurveda Integr Med. 2023;14(2): 100544. 10.1016/j.jaim.2022.100544. 35337710 10.1016/j.jaim.2022.100544 PMC10307684

[131] Zou Y, Ding W, Wu Y, Chen T, Ruan Z. Puerarin alleviates inflammation and pathological damage in colitis mice by regulating metabolism and gut microbiota. Front Microbiol. 2023. 10.3389/fmicb.2023.1279029. 37908541 10.3389/fmicb.2023.1279029 PMC10614640

[132] Yang L, Ma XY, Mu KX, Dai Y, Xia YF, Wei ZF. Galangin targets HSP90β to alleviate ulcerative colitis by controlling fatty acid synthesis and subsequent NLRP3 inflammasome activation. Mol Nutr Food Res. 2023;67(11):2200755. 10.1002/mnfr.202200755. 10.1002/mnfr.202200755 37002873

[133] Shen X, Chen H, Wen T, Liu L, Yang Y, Xie F, Wang L. A natural chalcone cardamonin inhibits necroptosis and ameliorates dextran sulfate sodium (DSS)-induced colitis by targeting RIPK1/3 kinases. Eur J Pharmacol. 2023;5(954): 175840. 10.3389/fmicb.2023.1279029. 10.3389/fmicb.2023.1279029 37302524

[134] Alharbi TS, Alshammari ZS, Alanzi ZN, Althobaiti F, Elewa MA, Hashem KS, Al-Gayyar MM. Therapeutic effects of genistein in experimentally induced ulcerative colitis in rats via affecting mitochondrial biogenesis. Mol Cell Biochem. 2023;21:1–4. 10.1007/s11010-023-04746-8. 10.1007/s11010-023-04746-8 37084167

[135] Cao X, Cao L, Zhang W, Lu R, Bian JS, Nie X. Therapeutic potential of sulfur-containing natural products in inflammatory diseases. Pharmacol Ther. 2020;1(216): 107687. 10.1016/j.pharmthera.2020.107687. 10.1016/j.pharmthera.2020.107687 32966837

[136] Shirazi KM, Sotoudeh S, Shirazi AM, Moaddab SY, Nourpanah Z, Nikniaz Z. Effect of N-acetylcysteine on remission maintenance in patients with ulcerative colitis: a randomized, double-blind controlled clinical trial. Clin Res Hepatol Gastroenterol. 2021;45(4): 101532. 10.1016/j.clinre.2020.08.010. 33067169 10.1016/j.clinre.2020.08.010

[137] Zhang Y, Guo Y, Zhang Q, Zhu Y, Xia Y, Wei Z, Dai Y. Diallyl trisulfide, a major bioactive constituent of garlic, promotes colonic mucosal healing in ulcerative colitis through accelerating focal adhesion assembly and consequent epithelial cell migration via the Rab21-Integrin β1-Fak pathway. Mol Nutr Food Res. 2023;20:2200784. 10.1002/mnfr.202200784. 10.1002/mnfr.202200784 36938915

[138] Lohning A, Kidachi Y, Kamiie K, Sasaki K, Ryoyama K, Yamaguchi H. 6- (methylsulfinyl) hexyl isothiocyanate (6-MITC) from *Wasabia japonica* alleviates inflammatory bowel disease (IBD) by potential inhibition of glycogen synthase kinase 3 beta (GSK-3β). Eur J Med Chem. 2021;15(216): 113250. 10.18632/oncotarget.22902. 10.18632/oncotarget.22902 33691258

[139] Zhang Y, Tan L, Li C, Wu H, Ran D, Zhang Z. Sulforaphane alter the microbiota and mitigate colitis severity on mice ulcerative colitis induced by DSS. AMB Express. 2020;10(1):119. 10.1186/s13568-020-01053-z. 32621086 10.1186/s13568-020-01053-z PMC7334330

[140] Pang L, Wang T, Liao Q, Cheng Y, Wang D, Li J, Fu C, Zhang C, Zhang J. Protective role of ergothioneine isolated from *Pleurotus ostreatus* against dextran sulfate sodium-induced ulcerative colitis in rat model. J Food Sci. 2022;87(1):415–26. 10.1111/1750-3841.15982. 34873706 10.1111/1750-3841.15982

[141] Maio AC, Basile G, Iacopetta D, Catalano A, Ceramella J, Cafaro D, Saturnino C, Sinicropi MS. The significant role of nutraceutical compounds in ulcerative colitis treatment. Curr Med Chem. 2022;29(24):4216–34. 10.2174/0929867329666211227121321. 34961429 10.2174/0929867329666211227121321

[142] Parian AM, Limnetic BN, Shah ND, Mullin GE. Nutraceutical supplements for inflammatory bowel disease. Nutr Clin Pract. 2015;30(4):551–8. 10.1177/0884533615586598. 26024677 10.1177/0884533615586598

[143] Shirazi KM, Nikniaz Z, Shirazi AM, Rohani M. Vitamin A supplementation decreases disease activity index in patients with ulcerative colitis: a randomized controlled clinical trial. Complement Ther Med. 2018;1(41):215–9. 10.1016/j.ctim.2018.09.026. 10.1016/j.ctim.2018.09.026 30477842

[144] Kondo K, Hiramoto K, Yamate Y, Goto K, Sekijima H, Ooi K. Ameliorative effect of high-dose vitamin C administration on dextran sulfate sodium-induced colitis mouse model. Biol Pharm Bull. 2019;42(6):954–9. 10.1248/bpb.b18-00967. 31155592 10.1248/bpb.b18-00967

[145] Qiu F, Zhang Z, Yang L, Li R, Ma Y. Combined effect of vitamin C and vitamin D3 on intestinal epithelial barrier by regulating Notch signaling pathway. Nutr Metab. 2021;18(1):49. 10.1186/s12986-021-00576-x. 10.1186/s12986-021-00576-x PMC8105975 33964955

[146] Guo X, Liu C, Huang Y. Efficacy and safety of vitamin d adjuvant therapy for ulcerative colitis: a meta-analysis. Comput Math Methods Med. 2022;20:2022. 10.1155/2022/6836942. 10.1155/2022/6836942 PMC9328974 35912148

[147] Liu KY, Nakatsu CH, Jones-Hall Y, Kozik A, Jiang Q. Vitamin E alpha-and gamma-tocopherol mitigate colitis, protect intestinal barrier function and modulate the gut microbiota in mice. Free Radical Biol Med. 2021;1(163):180–9. 10.1016/j.freeradbiomed.2020.12.017. 10.1016/j.freeradbiomed.2020.12.017 33352218

[148] Niu W, Chen X, Xu R, Dong H, Yang F, Wang Y, Zhang Z, Ju J. Polysaccharides from natural resources exhibit great potential in the treatment of ulcerative colitis: a review. Carbohyd Polym. 2021;15(254): 117189. 10.1016/j.carbpol.2020.117189. 10.1016/j.carbpol.2020.117189 33357839

[149] Yan S, Yin L, Dong R. Inhibition of IEC-6 cell proliferation and the mechanism of ulcerative colitis in C57BL/6 Mice by Dandelion root polysaccharides. Foods. 2023;12(20):3800. 10.3390/foods12203800. 37893693 10.3390/foods12203800 PMC10606498

[150] Feng W, Liu J, Tan Y, Ao H, Wang J, Peng C. Polysaccharides from *Atractylodes macrocephala* Koidz Ameliorate ulcerative colitis via extensive modification of gut microbiota and host metabolism. Food Res Int. 2020;138: 109777. 10.1016/j.foodres.2020.109777. 33288163 10.1016/j.foodres.2020.109777

[151] Batista JA, de Aguiar Magalhães D, Sousa SG, dos Santos FJ, Pereira CM, do Nascimento Lima JV, de Albuquerque IF, Bezerra NL, de Brito TV, da Silva Monteiro CE, Franco AX. Polysaccharides derived from *Morinda citrifolia* Linn. reduce inflammatory markers during experimental colitis. J Ethnopharmacol. 2020;10(248): 112303. 10.1016/j.jep.2019.112303. 10.1016/j.jep.2019.112303 31614204

[152] Zhang Y, Liu Y, Luo J, Liu Y, Yu S, Liu J. Rheum tanguticum polysaccharide alleviates DSS-induced ulcerative colitis and regulates intestinal microbiota in mice. Food Biosci. 2023;53: 102788. 10.1016/j.fbio.2023.102788. 10.1016/j.fbio.2023.102788

[153] Kye YJ, Lee SY, Kim HR, Lee BH, Park JH, Park MS, Ji GE, Sung MK. *Lactobacillus acidophilus* PIN7 paraprobiotic supplementation ameliorates DSS-induced colitis through anti-inflammatory and immune regulatory effects. J Appl Microbiol. 2022;132(4):3189–200. 10.1111/jam.15406. 34878713 10.1111/jam.15406

[154] Zhang C, Zhao Y, Jiang J, Yu L, Tian F, Zhao J, Zhang H, Chen W, Zhai Q. Identification of the key characteristics of *Bifidobacterium longum* strains for the alleviation of ulcerative colitis. Food Funct. 2021;12(8):3476–92. 10.1039/D1FO00017A. 33900330 10.1039/D1FO00017A

[155] Liu N, Wang H, Yang Z, Zhao K, Li S, He N. The role of functional oligosaccharides as prebiotics in ulcerative colitis. Food Funct. 2022;13(13):6875–93. 10.1039/D2FO00546H. 35703137 10.1039/D2FO00546H

[156] Wu A, Gao Y, Kan R, Ren P, Xue C, Kong B, Tang Q. Alginate oligosaccharides prevent dextran-sulfate-sodium-induced ulcerative colitis via enhancing intestinal barrier function and modulating gut microbiota. Foods. 2023;12(1):220. 10.3390/foods12010220. 36613442 10.3390/foods12010220 PMC9818813

[157] Chen W, Tan D, Yang Z, Tang J, Bai W, Tian L. Fermentation patterns of prebiotics fructooligosaccharides-SCFA esters inoculated with fecal microbiota from ulcerative colitis patients. Food Chem Toxicol. 2023;1(180): 114009. 10.1016/j.fct.2023.114009. 10.1016/j.fct.2023.114009 37652126

[158] Mota J, Casimiro S, Fernandes J, Hartmann RM, Schemitt E, Picada J, Costa L, Marroni N, Raymundo A, Lima A, Ferreira RB. Lupin protein concentrate as a novel functional food additive that can reduce colitis-induced inflammation and oxidative stress. Nutrients. 2022;14(10):2102. 10.3390/nu14102102. 35631241 10.3390/nu14102102 PMC9143369

[159] Zhang B, Xu Y, Zhao C, Zhang Y, Lv H, Ji X, Wang J, Pang W, Wang X, Wang S. Protective effects of bioactive peptides in foxtail millet protein hydrolysates against experimental colitis in mice. Food Funct. 2022;13(5):2594–605. 10.1039/D1FO02482E. 35166735 10.1039/D1FO02482E

[160] Xiang XW, Zhou XL, Wang R, Shu CH, Zhou YF, Ying XG, Zheng B. Protective effect of tuna bioactive peptide on dextran sulfate sodium-induced colitis in mice. Mar Drugs. 2021;19(3):127. 10.3390/md19030127. 33652919 10.3390/md19030127 PMC7996728

[161] Zhou Y, Wang D, Duan H, Zhou S, Guo J, Yan W. The potential of natural oils to improve inflammatory bowel disease. Nutrients. 2023;15(11):2606. 10.3390/nu15112606. 37299569 10.3390/nu15112606 PMC10255559

[162] Bartoszek A, Makaro A, Bartoszek A, Kordek R, Fichna J, Salaga M. Walnut oil alleviates intestinal inflammation and restores intestinal barrier function in mice. Nutrients. 2020;12(5):1302. 10.3390/nu12051302. 32370215 10.3390/nu12051302 PMC7284466

[163] Takashima T, Sakata Y, Iwakiri R, Shiraishi R, Oda Y, Inoue N, Nakayama A, Toda S, Fujimoto K. Feeding with olive oil attenuates inflammation in dextran sulfate sodium-induced colitis in rat. J Nutr Biochem. 2014;25(2):186–92. 10.1016/j.jnutbio.2013.10.005. 24445043 10.1016/j.jnutbio.2013.10.005

[164] Zhou Q, Ma L, Zhao W, Zhao W, Han X, Niu J, Li R, Zhao C. Flaxseed oil alleviates dextran sulphate sodium-induced ulcerative colitis in rats. J Funct Foods. 2020;64: 103602. 10.1016/j.jff.2019.103602. 10.1016/j.jff.2019.103602

[165] Park JS, Choi J, Hwang SH, Kim JK, Kim EK, Lee SY, Lee BI, Park SH, Cho ML. Cottonseed oil protects against intestinal inflammation in dextran sodium sulfate-induced inflammatory bowel disease. J Med Food. 2019;22(7):672–9. 10.1089/jmf.2018.4323. 31112045 10.1089/jmf.2018.4323

[166] Uchiyama K, Takami S, Suzuki H, Umeki K, Mochizuki S, Kakinoki N, Iwamoto J, Hoshino Y, Omori J, Fujimori S, Yanaka A. Efficacy and safety of short-term therapy with indigo naturalis for ulcerative colitis: an investigator-initiated multicenter double-blind clinical trial. PLoS ONE. 2020;15(11): e0241337. 10.1371/journal.pone.0241337. 33151988 10.1371/journal.pone.0241337 PMC7644062

[167] Papada E, Forbes A, Amerikanou C, Torović L, Kalogeropoulos N, Tzavara C, Triantafillidis JK, Kaliora AC. Antioxidative efficacy of a *Pistacia lentiscus* supplement and its effect on the plasma amino acid profile in inflammatory bowel disease: a randomised, double-blind, Placebo-Controlled Trial. Nutrients. 2018;10(11):1779. 10.3390/nu10111779. 30453494 10.3390/nu10111779 PMC6267573

[168] Baghizadeh A, Davati A, Heidarloo AJ, Emadi F, Aliasl J. Efficacy of Plantago major seed in management of ulcerative colitis symptoms: a randomized, placebo controlled, clinical trial. Complement Ther Clin Pract. 2021;1(44): 101444. 10.1016/j.ctcp.2021.101444. 10.1016/j.ctcp.2021.101444 34265576

